# Modification‐Selective Inhibition of Succinate Dehydrogenase a Enables Tumor‐Restricted Mitochondrial Modulation

**DOI:** 10.1002/advs.77046

**Published:** 2026-08-19

**Authors:** Kaikai Xu, Lulu Kong, Kebin Ye, Xinlu Bao, Haowen Liu, Ning Wang, Yongming Deng, Wei Wang, Shaohua Wei, Lin Zhou

**Affiliations:** ^1^ College of Chemistry and Materials Science Jiangsu Key Laboratory of Bio‐functional Materials Jiangsu Collaborative Innovation Centre of Biomedical Functional Materials Nanjing Normal University Nanjing China; ^2^ School of Science Key Laboratory of High Performance Scientific Computation Xihua University Chengdu China; ^3^ Department of Urology Nanjing Drum Tower Hospital the Affiliated Hospital of Nanjing University Medical School Institute of Urology Nanjing University Nanjing China; ^4^ School of Chemistry and Chemical Engineering Yancheng Institute of Technology Yancheng China

**Keywords:** non‐acetylated state, phthalocyanine, selective regulation, succinate dehydrogenase

## Abstract

Succinate dehydrogenase (SDH, complex II) connects the tricarboxylic acid (TCA) cycle to the electron transport chain (ETC), maintaining mitochondrial energy homeostasis. Selective SDH inhibition in diseased tissue has remained challenging due to the highly conserved catalytic architecture across normal tissues. Here, we reveal that SDH catalytic activity depends on the acetylation state of its catalytic subunit A (SDHA): the non‐acetylated form is catalytically active but exhibits fragile homeostasis in bladder cancer, contrasting with its robust maintenance in normal cells. Guided by this insight, we designed ZPI‐3, a conformationally optimized inhibitor that selectively engages the non‐acetylated SDHA, suppressing tumor SDH activity and growth while sparing normal cells. In orthotopic NMIBC models, ZPI‐3 outperforms mitomycin‐C. Differential SDH responses to ZPI‐3 in clinical specimens further suggest its potential for patient stratification and precision therapy. These findings establish modification‐selective inhibition as a paradigm for tumor‐selective mitochondrial modulation.

## Graphical abstract

This work identifies acetylation‐dependent SDHA homeostasis as a determinant of tumor‐specific metabolic vulnerability. Guided by this mechanism, a modification‐selective inhibitor, ZPI‐3, selectively targets non‐acetylated SDHA, achieving tumor‐restricted SDH inhibition and effective bladder cancer control. Differential responses in clinical specimens highlight its potential for patient stratification and precision mitochondrial therapy.

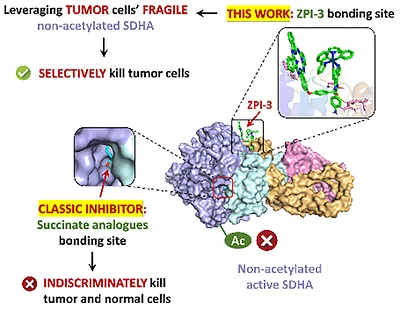

## 1 Introduction

Succinate dehydrogenase (SDH, mitochondrial complex II) uniquely connects the tricarboxylic acid (TCA) cycle with the electron transport chain (ETC), integrating metabolic flux with oxidative phosphorylation to sustain cellular energy homeostasis [1, 2, 3]. Conventional SDH inhibitors, such as succinate analogs that compete for the substrate‐binding site, lack selectivity between diseased and normal tissues because of the highly conserved catalytic architecture, often leading to systemic toxicity [4]. Hence, developing chemical strategies that exploit disease‐specific vulnerabilities beyond the active site remains a major unmet need [5, 6].

Here, we uncover a striking biological asymmetry in catalytic subunit A of SDH (SDHA) dependency: while normal cells rely on SDHA for proliferation and survival, bladder tumor cells remain viable upon its suppression. Although SDHA expression and basal SDH activity are comparable between normal and bladder tumor cells, the catalytically active, non‐acetylated form of SDHA exhibits robust homeostasis in normal cells but fragile maintenance in cancer [7, 8]. Such a difference reveals an opportunity for modification‐selective SDH inhibition to achieve tumor‐specific SDH inhibition [9, 10].

Guided by conformational optimization and structure‐based modeling, we identified a scaffold, ZPI‐3, whose spatial architecture complements a non‐canonical regulatory groove, enabling selectively engages the non‐acetylated, catalytically active form of SDHA for modification‐selective inhibition [11].

Unlike conventional substrate‐mimetic inhibitors, ZPI‐3 preferentially suppresses SDHA activity in tumor cells while exerting minimal effects on normal cells, thereby overcoming the long‐standing challenge of systemic toxicity. In orthotopic non‐muscle‐invasive bladder cancer (NMIBC) models, ZPI‐3 outperforms the clinically recommended mitomycin‐C (MMC) in tumor control, cure, and survival rates. Beyond SDH inhibitor, ZPI‐3 also acts as a photosensitizer, further improving therapeutic performance when combined with illumination. Our improved assay further reveals that assessing the differential effects of ZPI‐3 on SDH activity between clinical tumor and adjacent tissues provides a potential strategy to identify patients most likely to benefit, thereby establishing a framework for precision bladder cancer therapy (Scheme 1).

**SCHEME 1:**
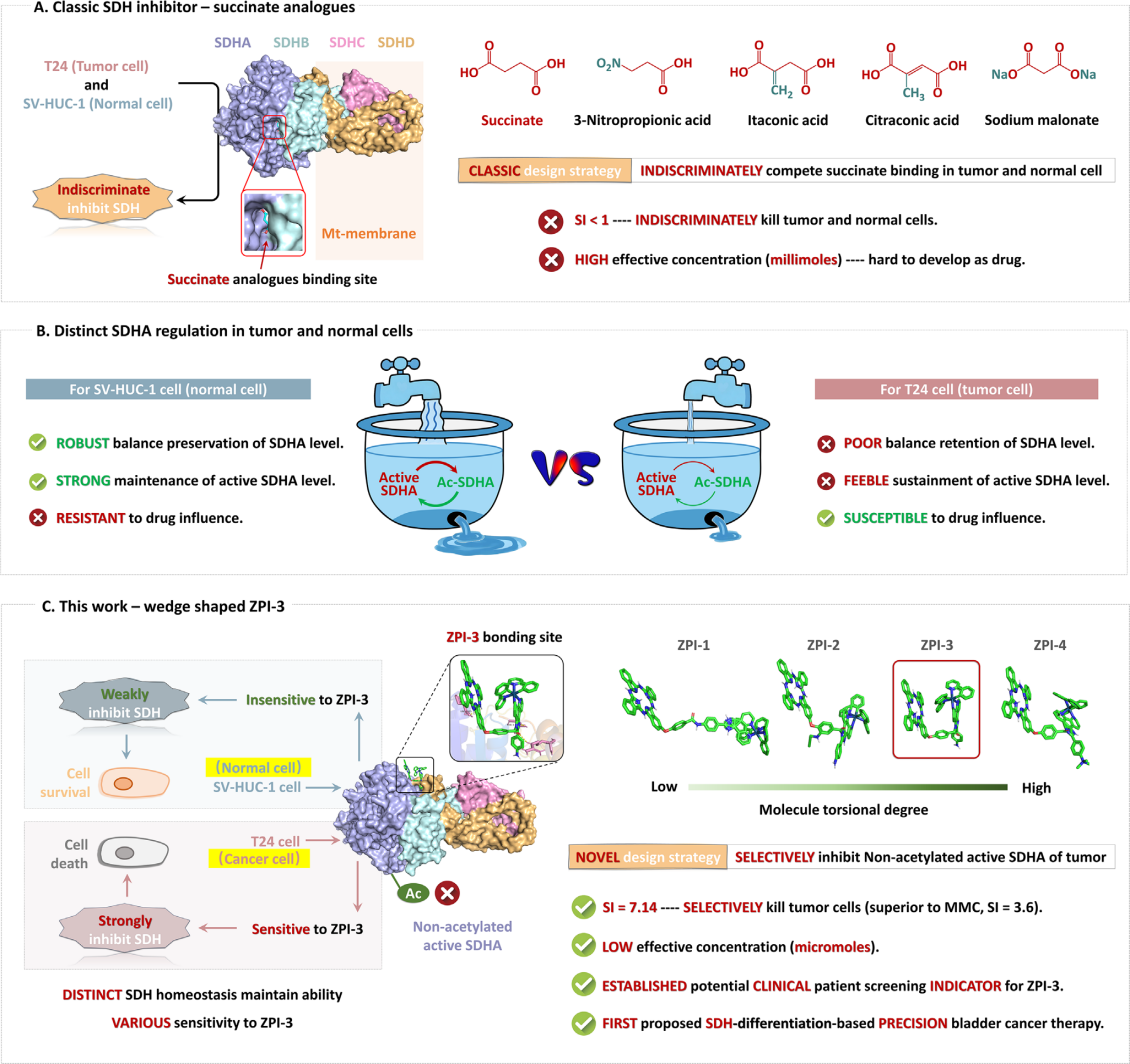
(A) Structural and application issues of classical SDH inhibitors, (B) Distinct SDHA regulation in tumor and normal cells, (C) A modification‐selective inhibitor, ZPI‐3, selectively targets non‐acetylated SDHA, achieving tumor‐restricted SDH inhibition and effective bladder cancer control.

Collectively, these findings establish a new chemical paradigm for SDH‐targeted intervention, revealing SDHA acetylation homeostasis as a determinant of cellular resilience to metabolic stress and offering a blueprint for selective modulation of mitochondrial metabolism in cancer.

## 2 Results and Discussion

ZnPc‐COOH is a photosensitizer that exhibits selective cytotoxicity toward bladder cancer cells (T24) while showing low toxicity to normal bladder epithelial cells (SV‐HUC‐1) under dark conditions. However, its selectivity index (SI = IC_50in SV‐HUC‐1_/IC_50in T24_) is only 2.4, which is lower than that of the clinically used chemotherapeutic agent MMC (SI = 3.6; Scheme S1, Figure S1 and Table S1) [12]. To enhance tumor selectivity, we introduced an iridium complex—known for its intrinsic antitumor activity—onto the ZnPc‐COOH scaffold, generating a series of four derivatives (ZPI‐1 to ZPI‐4) with progressively increased molecular distortion (Figure 1A, Schemes S2–S6).

**FIGURE 1:**
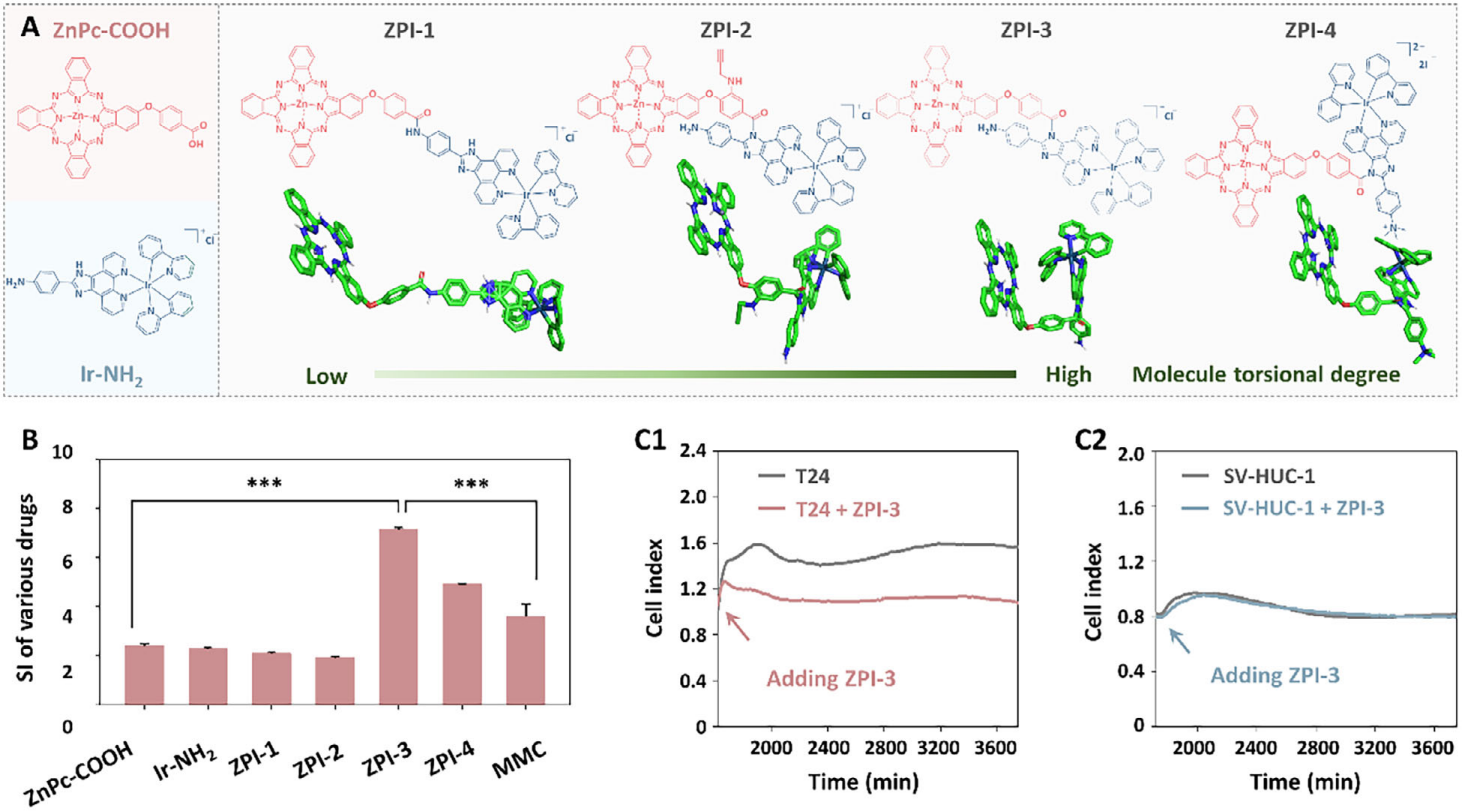
(A) Chemical structures of ZnPc‑COOH, Ir‑NH_2_, and ZPI‑1–ZPI‑4. (B) Selectivity indices (SI) of these compounds and MMC in SV‑HUC‑1 and T24 cells. Data are presented as the mean ± s.d. (*n*  =  3, each group). Group differences were analyzed using one‐way ANOVA followed by Tukey's multiple comparison correction. ^***^ *p* < 0.001. (C) Growth curves of T24 (C1) and SV‑HUC‑1 (C2) cells treated with ZPI‑3 (38.5 µM) for 3600 min.

The incorporation of the iridium complex markedly improved the safety profile of phthalocyanine toward SV‐HUC‐1 cells, with ZPI‐3 showing the best selectivity (SI = 7.14; Figure 1B, Figures S2 and S3) [13]. ZPI‐3 effectively inhibited the proliferation of T24 cells, while exhibiting negligible effects on SV‐HUC‐1 cells under identical conditions (Figure 1C1,C2). Furthermore, even in the absence of light irradiation, ZPI‐3 demonstrated potent cytotoxicity against other bladder cancer cell lines, including 5637 cells (dark IC_50_ = 11.3 µM) and murine bladder cancer MB49 cells (dark IC_50_ = 35 µM).

These results indicate that conjugating an iridium complex to the ZnPc‐COOH molecule is an effective strategy to enhance its safety toward normal cells [14]. Among the four derivatives with varying degrees of molecular distortion, ZPI‐3 displayed the highest selectivity index, exceeding that of MMC, and thus represents an optimized lead for further investigation.

To elucidate the basis of ZPI‐3's tumor‐selective cytotoxicity, we first examined its subcellular distribution and uptake. Confocal microscopy revealed predominant mitochondrial localization of ZPI‐3 in both T24 and SV‐HUC‐1 cells (Figure 2A, Figures S4 and S5), while quantitative analysis confirmed comparable intracellular accumulation in the two cell types (Figure 2B). These results excluded differential uptake or localization as the cause of tumor selectivity, prompting us to identify its molecular targets.

**FIGURE 2:**
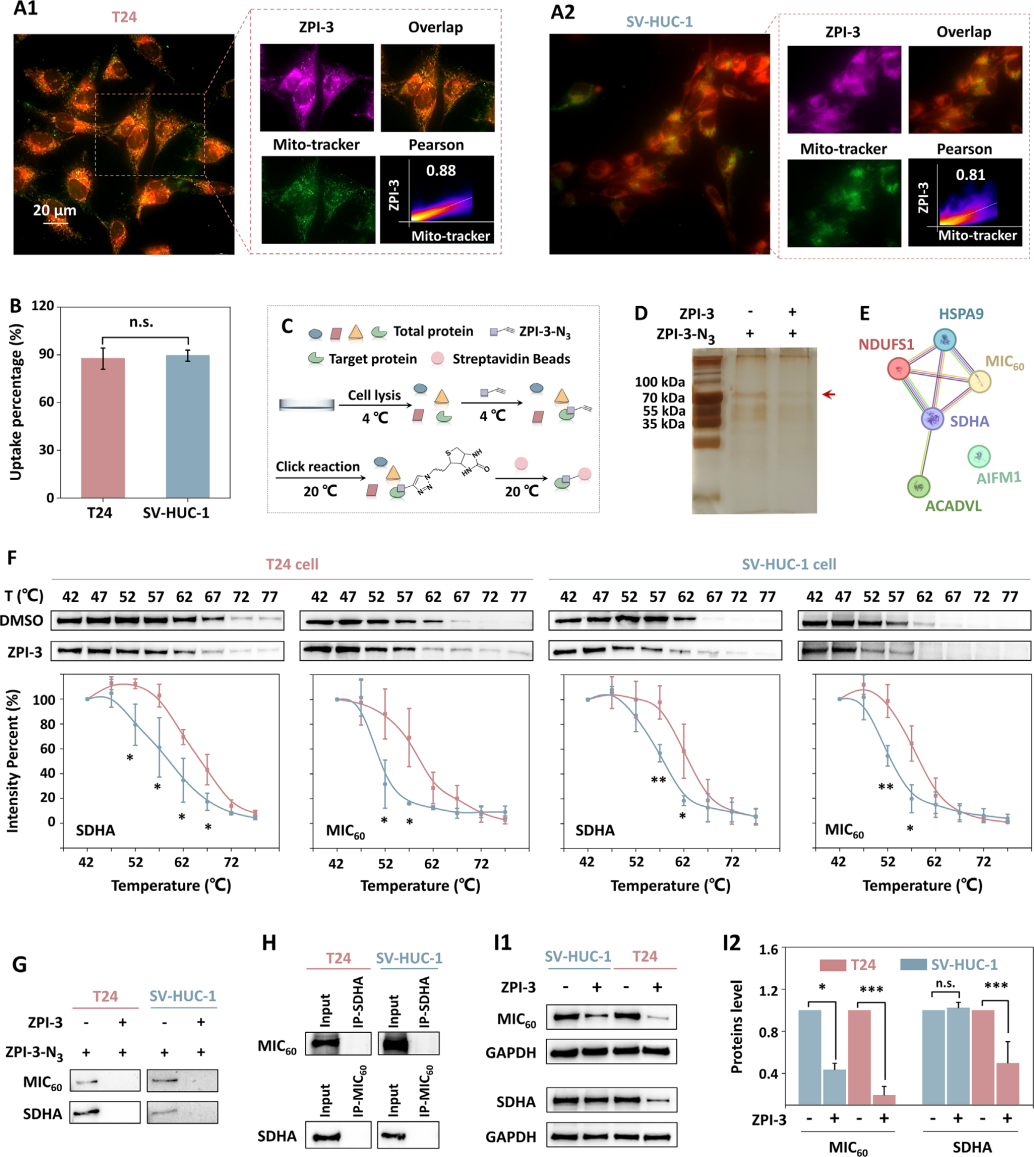
(A) Subcellular localization of ZPI‑3 (2.5 µM) for 24 h in T24 (A1) and SV‑HUC‑1 (A2) cells. (B) Cellular uptake of ZPI‑3 (10 µM) after 24 h of treatment in both cell lines. Group differences were analysed by the two‐tailed Student's *t‐*test. (C) Schematic of the pulldown workflow. (D) Silver staining of ZPI‑3‑N_3_ (5 µM) pull‐down and competition assays. (E) Protein–protein interaction network of candidate targets. (F) Thermal stability of SDHA and MIC_60_ in the presence of ZPI‑3 (10 µM). Group differences were analysed by the two‐tailed Student's *t‐*test. (G) Immunoblot validation of pulldown and competition assays. (H) Co‑immunoprecipitation of SDHA and MIC_60_. (I) Immunoblot (I1) and quantification (I2) of SDHA and MIC_60_ expression after ZPI‑3 (5 µM) treatment for 24 h. Group differences are analyzed by the two‐tailed Student's *t‐*test. All data are presented as the mean ± s.d. (*n*  =  3, each group). n.s., not significant, *p < 0.05, ^**^ *p* < 0.01, ^***^ *p* < 0.001.

To enable target identification while retaining antitumor activity, an alkyne‐functionalized probe (ZPI‐3‐N_3_) was synthesized (Scheme S7, Figure S6). Pull‐down enrichment of ZPI‐3–bound proteins (Figure 2C) followed by competitive binding, liquid chromatography–tandem mass spectrometry (LC–MS/MS), subcellular colocalization, immunoblotting, and thermal stability analyses identified several potential targets [15].

Preincubation with ZPI‐3 significantly attenuated the ∼70 kDa band in the ZPI‐3‐N_3_ pull‐down sample (Figure 2D and Figure S7), suggesting the principal binding protein lies within this molecular weight range. Based on LC–MS/MS results, candidate proteins were screened according to mitochondrial localization, molecular weight (60–80 kDa), and peptide score, yielding six candidates: MIC_60_, SDHA, HSPA9, ACADVL, NDUFS1, and AIFM1 (Figure 2E, Table S2). Thermal shift assays revealed that ZPI‐3 significantly altered the stability of MIC_60_ and SDHA in both T24 and SV‐HUC‐1 cells (Figure 2F), while no significant effect was observed for the remaining four proteins (Figures S8 and S9). Most CETSA studies have relied on ligand‐induced thermal stabilization as evidence of target engagement; however, it should be noted that certain compounds can also elicit thermal destabilization of their protein targets [16, 17, 18]. In this context, the classical SDH inhibitors 3‐nitropropionic acid (3‐NP) [8] and sodium malonate (SM) [2] exhibited differential effects on the thermal stability of SDHA (Figure S10). Furthermore, the isothermal dose–response CETSA (ITDR‐CETSA) experiment with ZPI‐3 and SDHA (Figure S11) provides direct evidence that the observed destabilization of SDHA in this study cannot be attributed merely to limitations of the experimental conditions. Moreover, preincubation of cell lysates with ZPI‐3 abolished the immunoblot signals of MIC_60_ and SDHA in pull‐down assays (Figure 2G), reinforcing these as direct binding partners [19].

Co‐immunoprecipitation (Co‐IP) excluded the possibility of a MIC_60–_SDHA complex (Figure 2H), indicating independent interactions with ZPI‐3. Western blot analysis showed comparable basal expression of MIC_60_ and SDHA between T24 and SV‐HUC‐1 cells. At 5 µM, ZPI‐3 downregulated MIC_60_ in both cell types but selectively reduced SDHA levels in T24 cells (Figure 2I). Collectively, these findings identify MIC_60_ and SDHA as the most probable targets of ZPI‐3, with SDHA modulation likely underlying its tumor‐selective cytotoxicity. Further validation of direct binding and functional contribution will clarify their respective roles in ZPI‐3 activity.

Knockdown of candidate target genes remains a classical approach for target validation [20]. Lentiviral transduction was used to generate T24 and SV‐HUC‐1 cells with varying levels of SDHA or MIC_60_ knockdown, as well as double‐knockdown lines (Table S3). The procedure itself did not alter proliferation or ZPI‐3 activity (Figures S12 and S13). In both T24 and SV‐HUC‐1 cells, knockdown of SDHA (Figure 3A,B), MIC_60_ (Figure S14), or simultaneous knockdown of both proteins (Table S3, Figures S15 and S16) resulted only in modest changes in sensitivity to ZPI‑3. These findings suggest that, under typical conditions, neither SDHA nor MIC_60_ is likely to represent a primary determinant of ZPI‑3 activity. Nevertheless, if the functional contribution of SDHA and MIC60 is governed not simply by their expression levels but by specific activity states, and if ZPI‑3 exerts differential effects on such states in distinct cellular contexts—or if the cellular dependence on these proteins varies substantially between the two models—they may still constitute critical targets mediating the activity of ZPI‑3.

**FIGURE 3:**
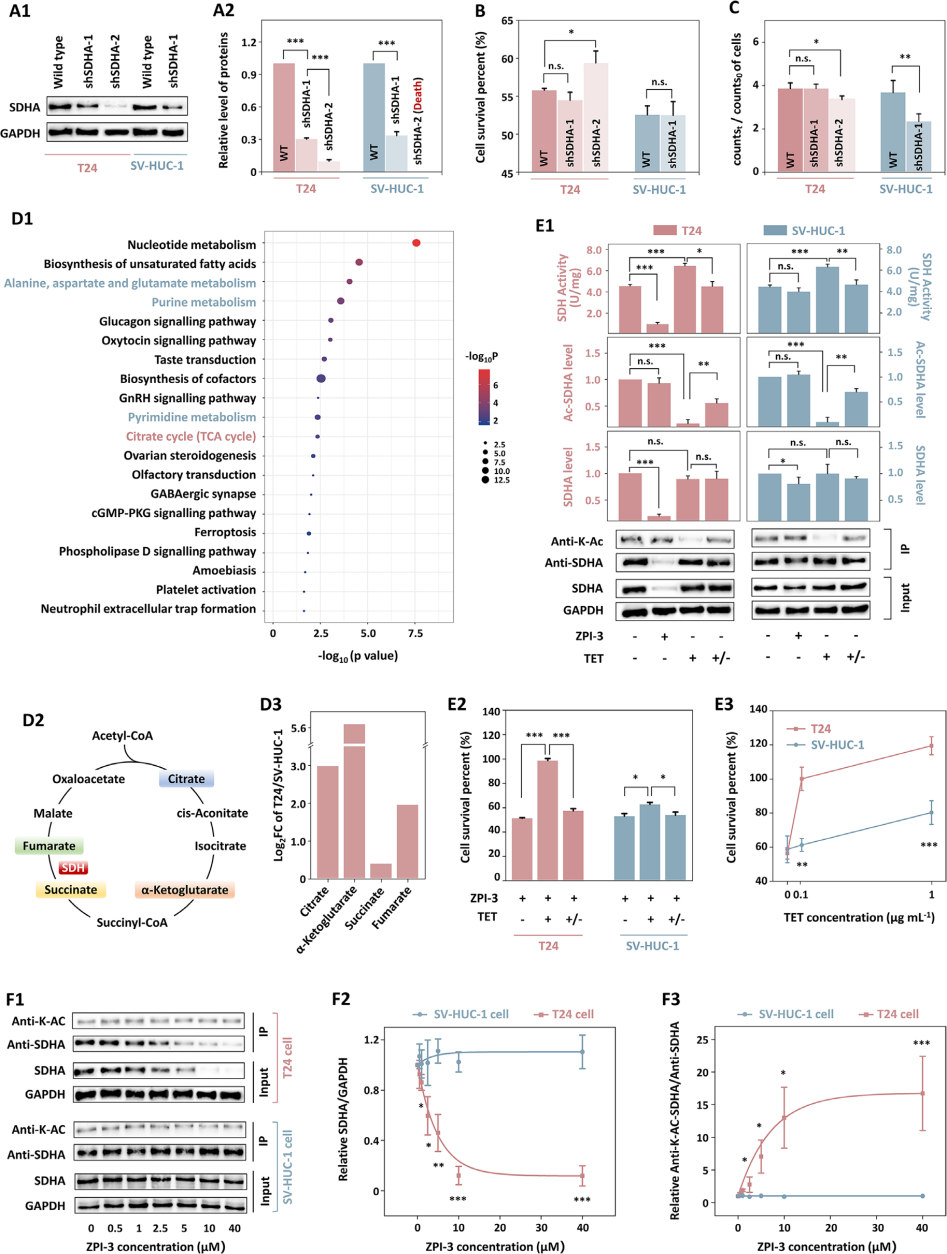
(A) Immunoblot (A1) and quantification (A2) of SDHA knockdown in T24 and SV‑HUC‑1 cells. (B) Effects of SDHA knockdown on cell proliferation. (C) Effects of SDHA knockdown on ZPI‑3 (38.5 µM) activity after 24 h of treatment. Group differences were analyzed using one‐way ANOVA followed by Tukey's multiple comparison correction. (D) Metabolomic KEGG analysis (D1), schematic of the TCA cycle (D2), and relative metabolite levels in T24 versus SV‑HUC‑1 cells (D3, *n* = 3). (E) Effects of TET on SDHA, Ac‑SDHA, and SDH activity (E1), on ZPI‑3 (38.5 µM) activity after 24 h of treatment (E2), and on sensitivity differences between cell lines (E3). Group differences were analysed using one‐way ANOVA followed by Tukey's multiple comparison correction. (F) Immunoblot analysis of SDHA and Ac‑SDHA levels after ZPI‑3 treatment with different concentrations (0, 0.5, 1, 2.5, 5, 10, 40 µM) after 24 h of treatment (F1) with quantification of the acetylation ratio (F2) and SDHA/GAPDH ratio (F3). Group differences were analyzed using the two‐tailed Student's *t‐*test. All data are presented as the mean ± s.d. (*n*  =  3, each group). n.s., not significant, ^*^ *p* < 0.05, ^**^ *p* < 0.01, ^***^ *p* < 0.001.

MIC_60_ is a central component of the mitochondrial inner membrane MICOS complex, essential for maintaining cristae architecture and thereby supporting oxidative phosphorylation [21]. Its functional integrity is closely linked to protein expression levels, and impairment of MIC_60_ has been reported to cause loss of crista junctions, onion‐like cristae structures, and compromised ATP synthesis. Consistent with these observations, T24 shMIC_60_‐2 cells exhibited such functional alterations (Figures S17 and S18), confirming that MIC_60_ knockdown leads to functional impairment and that its activity is indeed dependent on expression levels. Moreover, knockdown of MIC_60_ produced only subtle differences in cell proliferation between T24 and SV‐HUC‐1 cells (Figure S14A3,B3), indicating no fundamental divergence in MIC_60_ dependence between the two cell types. In parallel, ZPI‑3 reduced MIC_60_ protein levels in both T24 and SV‐HUC‐1 cells to a comparable extent (Figure 2I1,I2), suggesting that ZPI‑3 does not exert a differential effect on MIC_60_ across these cellular contexts. Taken together, these findings support the interpretation that MIC_60_ is unlikely to represent a critical target mediating the activity of ZPI‐3.

For SDHA, the degree of cellular dependence differed markedly between T24 and SV‐HUC‐1 cells. In T24 cells, SDHA knockdown exerted only a minor effect on proliferation, whereas in SV‐HUC‐1 cells, reduction of SDHA expression by 67% led to pronounced growth inhibition (Figure S19), and further depletion resulted in cell death (Figure 3A). In addition, ZPI‑3 elicited differential downregulation of SDHA protein levels in the two cell types (Figure 2I1,I2). Thus, although SDHA knockdown alone produced only modest changes in ZPI‑3 sensitivity, the strong cell‐type–specific dependence on SDHA, together with the differential regulation of its protein levels by ZPI‑3, suggests that SDHA may still represent a critical target contributing to the activity of ZPI‑3.

Metabolite analysis revealed higher TCA cycle flux and elevated levels of citrate, α‐ketoglutarate, succinate, and fumarate in T24 cells relative to SV‐HUC‐1 (Figure 3D, Table S4), despite comparable SDHA expression and SDH activity (Figures S20 and S21). This suggests that differences in SDH regulation, rather than abundance, underlie the observed selectivity [22].

Although succinate binding occurs at SDHA, even 80%–90% knockdown failed to significantly reduce SDH activity (Figure S21), consistent with prior reports that trace residual SDHA expression suffices to maintain ∼50% activity [9]. Thus, SDHA protein levels do not directly reflect functional activity. ZPI‐3 reduced SDHA and SDH activity in T24 cells without altering Ac‐SDHA levels, whereas SV‐HUC‐1 cells remained unaffected (Figure 3E1). Thus, we propose that ZPI‐3 preferentially targets the non‐acetylated active form of SDHA. SIRT3 serves as a key regulator of SDHA acetylation, suppressing its modification and thereby maintaining enzymatic activity. Myc, through activation of SKP2, promotes degradation of SIRT3, which in turn facilitates SDHA acetylation and functional inactivation [9, 23]. Tetracycline (TET) has been shown to reduce SDHA acetylation levels by inhibiting Myc activity. Although additional downstream effects of Myc may also be influenced by tetracycline, the feasibility of tetracycline as an inhibitor of SDHA acetylation has been experimentally validated [9]. TET transiently decreased SDHA acetylation and enhanced SDH activity; under these conditions, ZPI‐3 activity in T24 cells was nearly abolished but fully restored upon TET withdrawal, while SV‐HUC‐1 cells remained resistant (Figure 3E1–E3) [23]. Increasing ZPI‐3 concentrations progressively reduced total SDHA and the fraction of non‐acetylated SDHA in T24 cells, but not in SV‐HUC‐1 (Figure 3F1–F3) [24].

Microscale thermophoresis (MST) is a widely applied technique for demonstrating drug–protein interactions. Analysis revealed that SDHA binds to ZPI‑3 with a dissociation constant (K_d_) of 42 nM, indicating high‐affinity binding (Figure S22). Within intact cells, where both enzymatic systems and signaling pathways coexist, the effect of ZPI‑3 on non‐acetylated SDHA could theoretically arise either from direct target engagement or from indirect downstream regulation. In contrast, cell lysates retain enzymatic systems but lack active signaling pathways, thereby providing a simplified context for target validation. In T24 cell lysates, addition of a broad‐spectrum deacetylase inhibitor mixture markedly reduced SDH activity, confirming the presence of enzymatic machinery mediating SDHA deacetylation and demonstrating that this process can be blocked pharmacologically. Without inhibitors, incubation with 5 µM ZPI‑3 for 30 min decreased SDH activity by 46.7%; however, when deacetylation was restricted—thus lowering the abundance of non‐acetylated SDHA—the inhibitory effect of ZPI‑3 was reduced to 10.6%. These findings argue against an exclusively indirect regulatory mechanism of ZPI‑3 on SDH activity (Figure S23). The same pattern was reproduced in lysates from T24 shSDHA‑2 cells, supporting the conclusion that SDH activity is primarily determined by the abundance of non‐acetylated SDHA (Figure S23). In SV‐HUC‐1 and SV‐HUC‐1 shSDHA‑1 lysates, deacetylase inhibitors similarly suppressed SDH activity; however, ZPI‑3 exerted only weak inhibitory effects regardless of inhibitor treatment (Figure S23), suggesting that SV‐HUC‐1 cells possess a more robust compensatory mechanism for maintaining SDHA homeostasis.

Together, these results prove that the non‐acetylated active form of SDHA could as the critical mediator of ZPI‐3. The differential capacity of T24 and SV‐HUC‐1 cells to maintain SDHA homeostasis or resist perturbation underlies their divergent responses. Genomic analyses revealed significant differences in genes related to SDHA acetylation, SDH subunits, and complex assembly factors (Figure S24), which may explain the distinct homeostatic capacities. Further studies are required to delineate the precise mechanisms [3].

We next examined why ZPI‐3 exhibits stronger tumor‐selective cytotoxicity than other ZPIs. Pre‐incubation with ZnPc‑COOH did not interfere with the enrichment of SDHA by ZPI‑3‑N_3_, whereas pre‐incubation with ZPI‑3 markedly reduced the ability of ZPI‑3‑N_3_ to enrich SDHA (Figure S25). These results indicate that the binding of ZPI‑3 to SDHA depends on the integrity of the full molecular structure of ZPI‑3. MTT and CCK‐8 assays gave markedly different results (Figure S1). Since MTT reduction mainly depends on SDH activity, whereas CCK‐8 reflects total dehydrogenase activity (Figure 4A1), the MTT assay also reports SDH function [25]. At one‐tenth of its IC_50_, only ZPI‐3 significantly reduced MTT conversion in T24 cells in a dose‐dependent manner (Figures 4A2–A4), suggesting direct interference with SDH activity.

**FIGURE 4:**
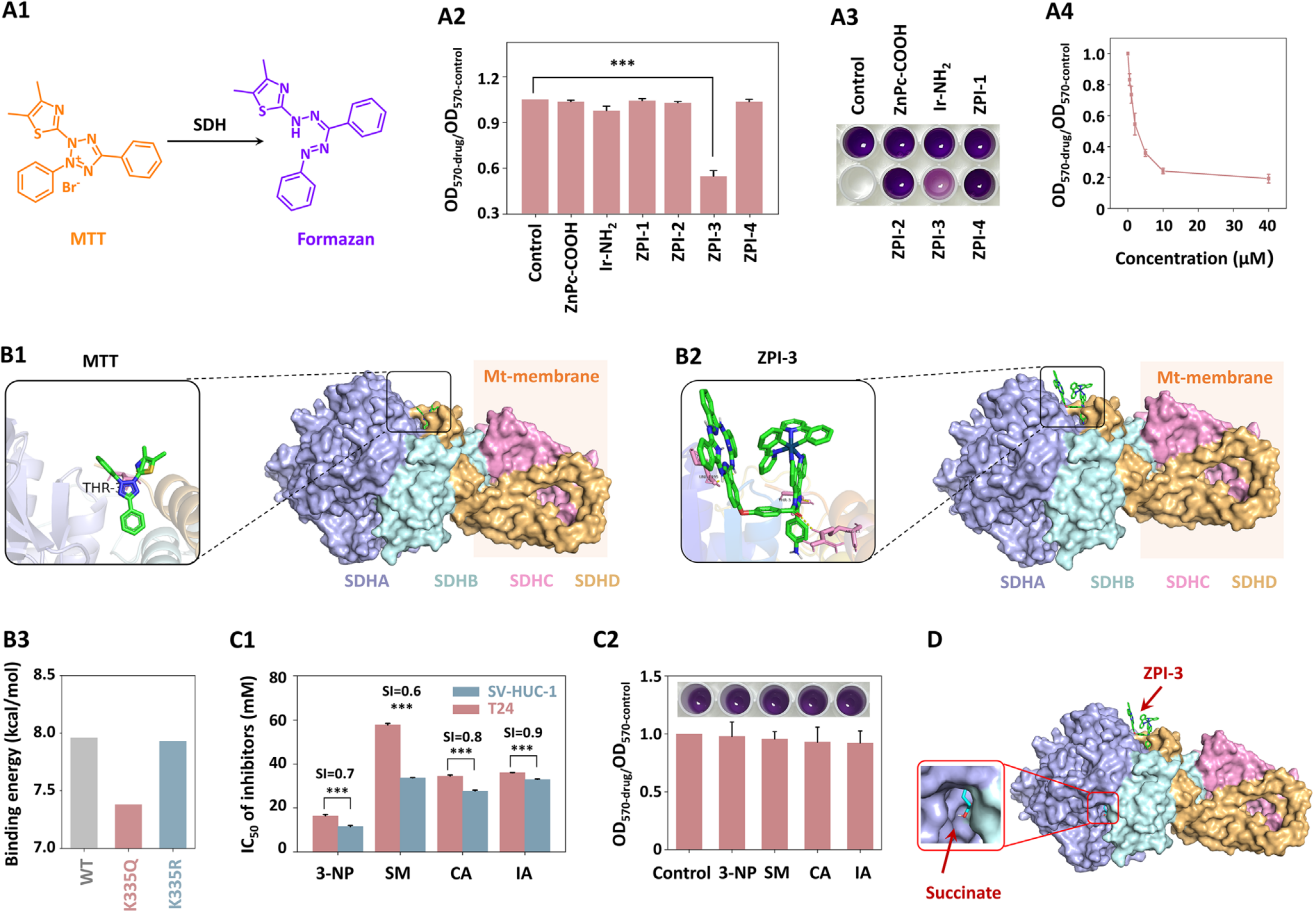
(A) Mechanism of MTT reduction by SDH (A1); effects of drugs at sub‑IC_50_ concentrations on MTT conversion (A2, A3). Data are presented as the mean ± s.d. (*n*  =  3, each group). Group differences were analysed using one‐way ANOVA followed by Tukey's multiple comparison correction. ^***^ *p* < 0.001; concentration‑dependent inhibition of MTT reduction by ZPI‑3 (A4). (B) Docking of MTT (B1) and ZPI‑3 (B2) to SDHA; binding energies of ZPI‑3 with WT, K335R, and K335Q SDHA (B3). (C) IC_50_ values of classical SDH inhibitors (3‑NP, IA, CA, SM) in SV‑HUC‑1 and T24 cells (C1), Data are presented as the mean ± s.d. (*n*  =  3, each group). Group differences are analyzed by the two‐tailed Student's *t*‐test. ^***^ *p* < 0.001; effects of these inhibitors on MTT conversion at sub‑IC_50_ concentrations (C2). (D) Comparison of succinate and ZPI‑3 binding sites on SDHA.

Molecular docking revealed that ZPI‐3 binds SDHA at the MTT‐binding pocket, inserting into a concave groove like a wedge (Figure 4B1,B2). Other ZPIs docked at distinct regions (Figure S26), indicating that ZPI‐3's geometry enables specific stabilization within this site. Previous reports used mass spectrometry and site‑directed mutagenesis studies to identify lysine 335 (K335) as the key functional acetylation site that determines SDHA enzymatic activity; acetylation at K335 directly inhibits SDH complex activity, which is highly conserved and widely validated across cellular systems [2, 9]. Mutation analysis further showed that ZPI‐3 binding energy to wild‐type and acetylation‐resistant SDHA (K335R) was higher than the acetylation‐mimetic mutant (K335Q) (Figure 4B3), proposing its affinity for the non‐acetylated, active form of SDHA [11].

Classical SDH inhibitors such as 3‐nitropropionic acid, itaconic acid, citraconic acid, and sodium malonate (Figure 4C1 and Figure S27) competitively block succinate binding but exhibit high IC_50_ values (tens of millimolar) and no tumor selectivity (SI < 1, Figure 4C2). At sub‐toxic concentrations, they do not affect MTT conversion (Figure 4D), confirming a distinct mechanism from ZPI‐3.

Based on the above findings, we propose the hypothesis that ZPI‑3 may influence SDH activity by binding at a site distinct from that of classical SDH inhibitors. However, docking results can only provide preliminary insights and should be regarded as suggestive rather than conclusive. Definitive identification of the in vivo binding site will require future validation through more precise and rigorous approaches, such as structural biology or biophysical analyses.

ZPI‐3 also acts as a photosensitizer with potential for photodynamic therapy (PDT) [26]. We next examined whether its effects on SDHA and MIC_60_ could synergize with PDT to enhance antitumor efficacy. MIC_60_, a key component of the mitochondrial inner membrane organizing system (MICOS), maintains cristae integrity, while SDH resides on the cristae, catalyzing the oxidation of succinate to fumarate and serving as complex II in the respiratory chain (Figure S28A1) [27]. Therefore, impairment of MIC_60_ may exert a potential auxiliary role in the activity of ZPI‑3.

Transmission electron microscopy revealed that ZPI‐3 caused marked cristae disruption and morphological changes in T24 cells, whereas these effects were much milder in SV‐HUC‐1 cells (Figure S28A2). Metabolite analysis further showed that ZPI‐3 blocked the conversion of succinate to fumarate, leading to succinate accumulation and reduced levels of other TCA metabolites in T24 cells. In contrast, SV‐HUC‐1 cells maintained metabolic balance through compensatory SDHA homeostasis (Figure S28B1) [28]. Consistently, ZPI‐3 induced significant mitochondrial depolarization and ATP depletion in T24 cells but had minimal effects in SV‐HUC‐1 (Figures S28B2,B3, Table S4). In T24 cells, ZPI‑3 induced significantly greater increases in both mitochondrial ROS (mtROS) and total ROS compared with SV‑HUC‑1 cells (Figure S28B4,B5).

Given the tight coupling between metabolism and redox regulation, we explored whether ZPI‐3‐induced metabolic stress sensitized T24 cells to oxidative damage. KEGG analysis revealed altered glutathione metabolism in both cell lines (Figure 5A and Figure S29). Even at a sevenfold higher concentration, ZPI‐3 caused a more pronounced reduction of GSH levels and GSH/GSSG ratio in T24 cells than in SV‐HUC‐1 (Figure 5B), accompanied by greater sensitivity to H_2_O_2_‐induced oxidative injury (Figure 5C). These results suggest that ZPI‐3 selectively impairs the antioxidant defence of T24 cells, potentially enhancing PDT efficacy while sparing normal cells [29].

**FIGURE 5:**
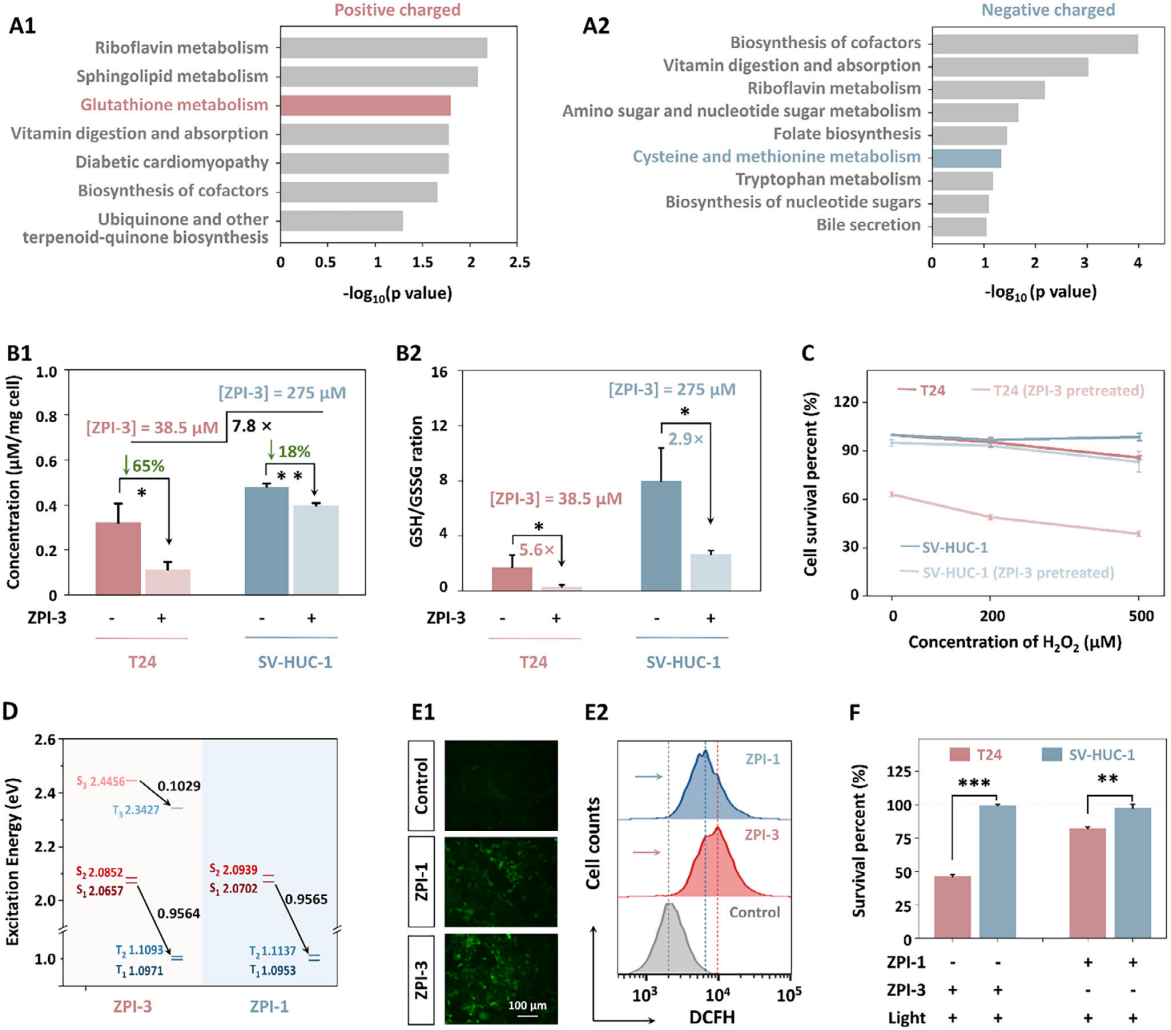
(A) KEGG pathway enrichment of ZPI‑3–treated T24 cells in positive (A1) and negative (A2) ion modes. (B) Effects of ZPI‑3 on GSH levels (B1) and GSH/GSSG ratios (B2) in T24 and SV‑HUC‑1 cells after 24 h of treatment at different concentrations. Group differences are analyzed by the two‐tailed Student's *t*‐test. (C) Differential oxidative stress responses after ZPI‑3 treatment. (D) Excited‑state energy diagrams of ZPI‑3 and ZPI‑1. (E) ROS generation by ZPI‑3 (7.5 nM) and ZPI‑1 (7.5 nM) in T24 cells measured by fluorescence microscopy (E1) and flow cytometry (E2); light dose = 12 J/cm^2^. (F) Selective cytotoxicity of ZPI‑3 and ZPI‑1 toward T24 cells ([ZPIs] = 7.5 nM; light dose = 12 J/cm^2^). Group differences are analyzed by the two‐tailed Student's *t*‐test. All data are presented as the mean ± s.d. (*n*  =  3, each group). n.s., not significant, ^*^ *p* < 0.05, ^**^ *p* < 0.01, ^***^ *p* < 0.001.

In PDT, intersystem crossing (ISC) from singlet to triplet states governs reactive oxygen (ROS) generation (Figure S30) [30]. DFT calculations showed that ZPI‐3 exhibits a small ISC energy gap (S_3_→T_3_, 0.1029 eV), favouring triplet population, while ZPI‐1 displays a much larger gap (S_1_→T_2_, 0.9569 eV) (Figure 5D). Consistent with this, ZPI‐3 displayed weaker fluorescence, lower charge‐transfer resistance, and stronger photocurrent—properties conducive to efficient ROS formation. Accordingly, ZPI‐3 produced more ROS than ZPI‐1 in both aqueous and cellular environments (Figure 5E and Figure S31). Under PDT irradiation, ZPI‐3 retained its tumor selectivity and exhibited markedly enhanced cytotoxicity toward T24 cells compared with ZPI‐1 (Figure 5F).

Collectively, these findings demonstrate that the molecular distortion of ZPIs governs both SDHA binding and PDT activity. Among them, ZPI‐3 achieves synergistic antitumor potency under PDT activation while maintaining excellent safety toward normal bladder epithelial cells.

NMIBC accounts for 75%–80% of bladder cancer cases [31]. To evaluate the in vivo antitumor efficacy of ZPI‐3 and its impact on recurrence, we established an orthotopic NMIBC model in C57BL/6 mice using mCherry‐labeled MB49 cells (Figure S32). Changes in intravesical mCherry fluorescence were used to monitor tumor progression and anticancer response [32].

Primary mouse bladder epithelial cells exhibited higher SDHA and acetylated SDHA (Ac‐SDHA) levels than MB49 and mCherry‐MB49 cells, although the Ac‐SDHA/SDHA ratios were comparable among them (Figure 6A1–A3, Figures S33, and S34). SDH activity varied across bladder tissues and cell types; notably, ZPI‑3 selectively suppressed SDH activity in tumor cells, while sparing normal epithelial cells and whole bladder tissues (Figure 6A4). This selective inhibition suggests a potential antitumor effect with a favorable safety profile in the orthotopic NMIBC model.

**FIGURE 6:**
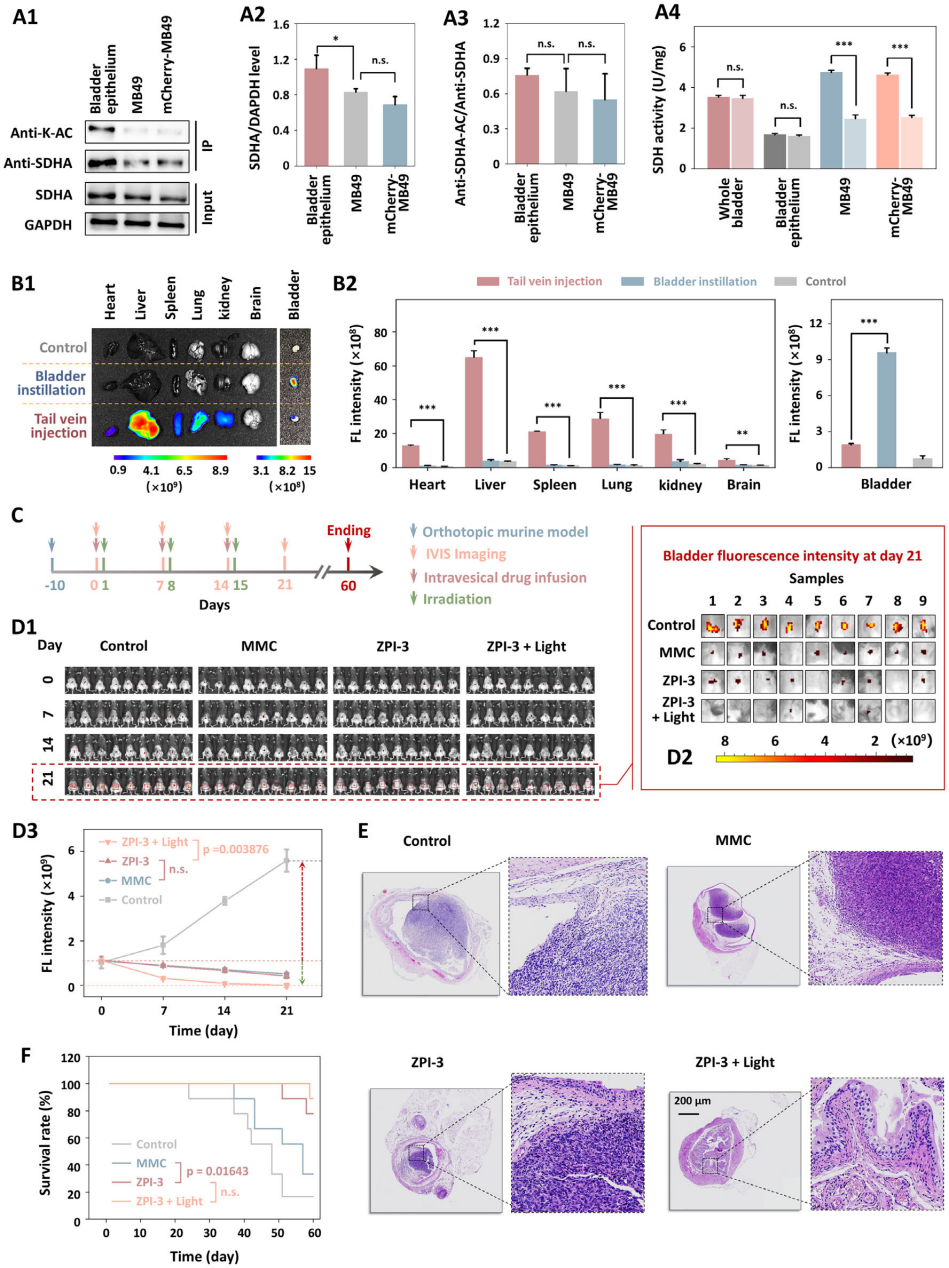
(A) Immunoblot of SDHA and Ac‑SDHA in primary bladder epithelial cells, MB49, and mCherry‑MB49 cells (A1), with quantification of SDHA (A2), Ac‑SDHA ratio (A3), and SDH activity following 24 h of treatment with or without ZPI‐3 (38.5 µM) (A4). Data are presented as the mean ± s.d. (*n* = 3, each group). Statistical analysis for Figure 6A2 and A3 was performed using one‐way ANOVA followed by multiple comparisons, while a two‐tailed Student's *t*‐test was used for Figure 6A4. (B) Biodistribution of ZPI‑3 (0.3125 mg/kg; light dose = 96 J/cm^2^) after intravesical instillation or intravenous injection: fluorescence images (B1), organ intensity comparison (B2), and bladder retention (B3). Data are presented as the mean ± s.d. (*n*  =  3, each group). Group differences were analyzed using a two‐tailed Student's *t*‐test. n.s., not significant, ^*^ *p* < 0.05, ^**^ *p* < 0.01, ^***^ *p* < 0.001. (C) Schematic of in vivo experimental design. (D) In vivo mCherry fluorescence imaging during 21 day treatment (D1), representative images at day 21 (D2), and tumor fluorescence intensity over time (D3). (E) H&E‑stained bladder sections from each treatment group. (F) Survival curves of treated mice over 60 days. All animal experimental data are presented as the mean ± s.d. (*n*  =  9, each group). Group differences were analysed using one‐way ANOVA followed by multiple comparisons. n.s., not significant, ^*^ *p* < 0.05, ^**^ *p* < 0.01.

For NMIBC treatment, both intravenous and intravesical administrations are clinically feasible [33]. Pharmacokinetic analysis revealed that intravesical delivery of ZPI‐3 minimized systemic distribution while maintaining prolonged bladder retention (Figure 6B). Therefore, intravesical instillation was employed for subsequent in vivo therapy. After confirming the safety of the administered dose (Figures S35–S38) and verifying that external light sources can effectively penetrate tissue to reach the mouse bladder and activate ZPI‑3 (Figure S39), we performed 21 day anticancer study and 60 day survival studies in mice NMIBC model using MMC, a first‐line clinical chemotherapeutic agent, as a positive control (Figure 6C).

Fluorescence imaging showed that average bladder fluorescence intensity in the control group increased 5.4‐fold by day 21, indicating tumor progression. In contrast, the MMC, ZPI‑3, and ZPI‑3 plus Light treatment groups exhibited fluorescence reductions of 52.9%, 62.6%, and 99.6%, respectively (Figure 6D), with the ZPI‑3 plus light group demonstrating the most pronounced antitumor effect (Figure S40). Histological analysis revealed dense tumor infiltration in the control group, partial tumor regression in the MMC and ZPI‐3 groups, and complete tumor clearance in the ZPI‐3 + Light group (Figure 6E).

Consistent with these results, the ZPI‐3 group exhibited a higher 60‐day survival rate than the MMC group, and PDT co‐treatment further improved survival outcomes (Figure 6F). Collectively, these results demonstrate that, under the experimental conditions of this study, ZPI‑3 exhibited superior antitumor efficacy compared with MMC in the orthotopic NMIBC mouse model. Moreover, when combined with photodynamic therapy, the antitumor activity of ZPI‑3 was further enhanced.

All antitumor agents exhibit patient‑specific applicability [34]. Our data indicate that the differential effect of ZPI‐3 on SDH catalytic activity in tumor versus normal tissue may serve as a feasible biomarker for patient selection. Transurethral resection of bladder tumor (TURBT) followed by intravesical chemotherapy is the guideline‑recommended treatment strategy for NMIBC [35, 36]. The resection margin in TURBT typically encompasses the tumor together with 1–2 cm of adjacent normal tissue. Building on this clinical context, we preliminarily hypothesize that comparing the differential effects of ZPI‑3 on SDH activity between tumor and peritumoral tissues may provide a potential basis for identifying patient subgroups most likely to benefit from ZPI‑3 therapy.

While SDH activity in Figure 3 was measured using a commercial kit, we optimized the protocol for application to ZPI‐3 sensitivity to cell and tissue lysates (Figure 7A) [37]. The modified assay yielded results consistent with the standard method in T24 and SV‐HUC‐1 cells, as well as in SDHA knockdown lines, confirming accuracy and independence from SDHA expression levels (Figure 7B).

**FIGURE 7:**
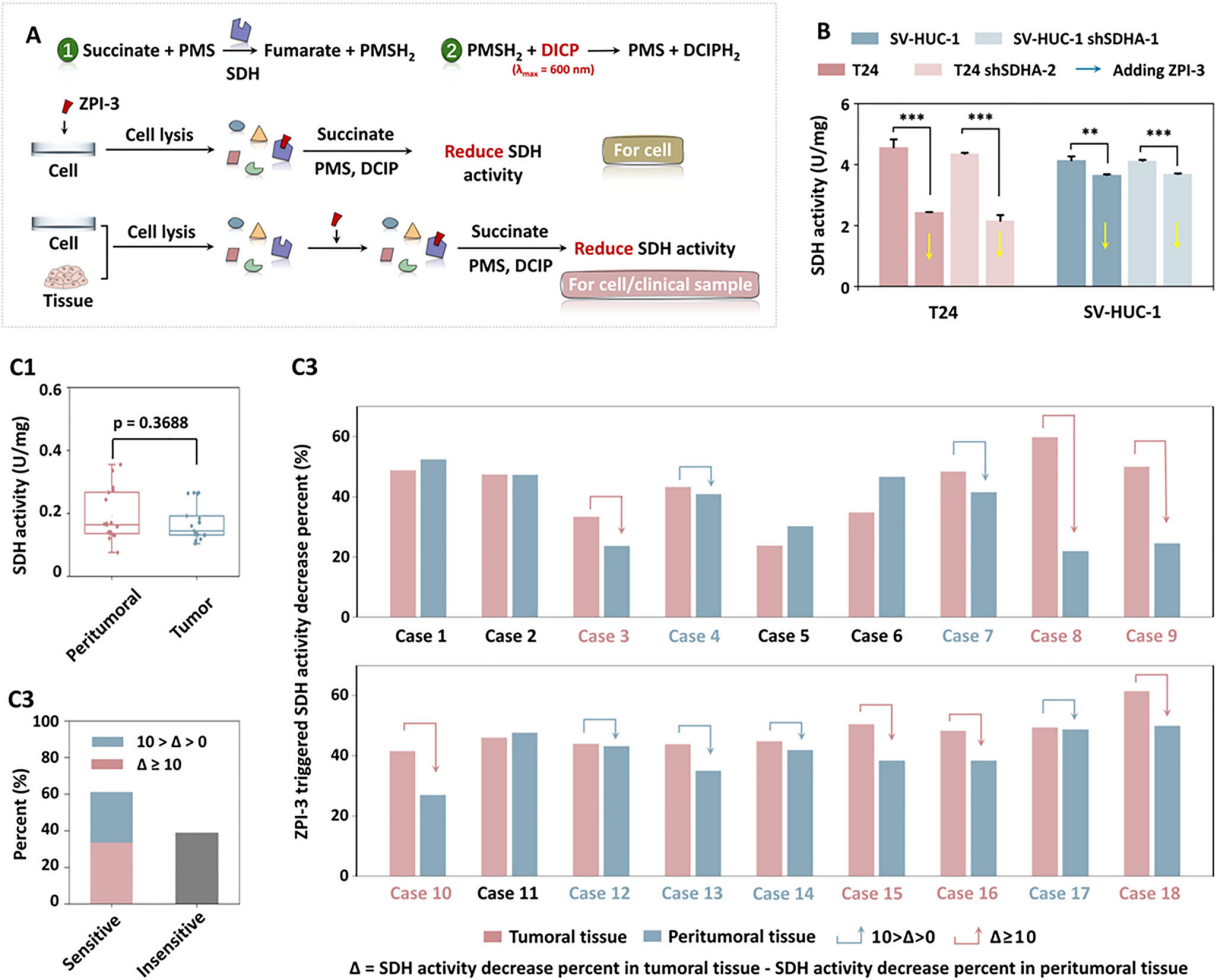
(A) Principle of the SDH activity assay, with comparison of the standard protocol and the optimized method suitable for evaluating ZPI‑3 effects in clinical samples. (B) Validation of the optimized assay: effects of ZPI‑3 (38.5 µM) on SDH activity in T24, T24 shSDHA‑2, SV‑HUC‑1, and SV‑HUC‑1 shSDHA‑1 cells after 30 min of treatment. Data are presented as the mean ± s.d. (*n*  =  3, each group). Group differences are analyzed by the two‐tailed Student's *t*‐test. ^**^ *p* < 0.01, ^***^ *p* < 0.001.(C) Clinical validation in NMIBC: comparison of SDH activity between tumor and peritumoral tissue (C1), Data are presented as the mean ± s.d. (*n*  =  18, each group). Group differences are analyzed by the two‐tailed Student's *t*‐test; differential effects of ZPI‑3 (38.5 µM) on paired tumor versus peritumoral tissue after 30 min of ex vivo treatment (C2); proportion of patients classified as sensitive (greater inhibition in tumor) or insensitive (greater inhibition in peritumoral tissue) (C3).

As shown in Figure 7C1, SDH activity did not differ significantly between tumor and peritumoral tissues from 18 NMIBC patients; in both tissue types, SDH activity could be suppressed by ZPI‑3. Pairwise comparison of each matched sample (Figure S41) revealed that in 13 of the 18 pairs (72.2%), ZPI‑3 reduced SDH activity more strongly in tumor tissue than in the corresponding peritumoral tissue (Figure 7C2,C3). Notably, in 7 pairs (38.9%), the reduction in tumor tissue exceeded that in peritumoral tissue by ≥ 10%. These findings suggest that, in a subset of patients, tumor SDH may be more responsive to ZPI‑3 than peritumoral SDH, and the relatively lower impact on peritumoral tissue could indicate a favorable safety potential. It is important to emphasize, however, that this study has clear limitations: first, the clinical sample size was small; second, all tissue validation experiments were performed in ex vivo lysate systems, where accuracy depends on tissue separation and identification, and is closely linked to the operator's expertise. Therefore, the current evidence should be regarded as preliminary proof‑of‑concept and is insufficient to support patient stratification or therapeutic decision‑making at this stage.

Tumor tissues exhibit considerable complexity and heterogeneity, with malignant cells often comprising multiple subtypes. To further explore this aspect, we examined the activity of ZPI‑3 in two additional bladder cancer cell lines (5637 and UM‑UC‑3). Unexpectedly, ZPI‑3 displayed antitumor activity and SDH suppression in 5637 cells comparable to that observed in T24 cells. In contrast, UM‑UC‑3 cells exhibited resistance to ZPI‑3 similar to SV‑HUC‑1 cells, accompanied by a comparable dependence on SDHA (Figures S42 and S43). These contrasting findings highlight the intrinsic heterogeneity of bladder cancer and underscore the importance of defining molecular characteristics as potential biomarkers to predict sensitivity to SDHA activity modulation.

To date, no studies have identified SDHA as a therapeutic target or precision medicine biomarker in bladder cancer (Figure S44). However, both published literature and our findings indicate that differences between bladder cancer cells and normal cells are not limited to SDHA expression levels, but extend to deeper regulatory mechanisms associated with acetylation. This distinction may provide an opportunity for drug development and represents a potential new strategy for precision therapy in NMIBC. Nevertheless, the specific molecular features that could serve as predictive biomarkers of sensitivity to SDHA activity modulation remain undefined. Future progress will depend not only on the discovery of such biomarkers but also on large‑scale clinical analyses to determine how these molecular characteristics influence patient outcomes, including prognosis, survival, and recurrence.

## 3 Conclusion

In summary, this work establishes acetylation‐dependent SDHA regulation as a determinant of mitochondrial metabolic vulnerability and introduces modification‐selective inhibition as a new chemical principle for SDH‐targeted therapy. By selectively engaging the non‐acetylated, catalytically active SDHA, ZPI‐3 achieves tumor‐restricted SDH inhibition and effective control of non‐muscle‐invasive bladder cancer with minimal toxicity (Figure S45). The discovery that acetylation homeostasis governs SDH resilience provides a mechanistic basis for tumor selectivity and opens new directions for exploiting post‐translational modifications to achieve metabolic precision in cancer treatment. Furthermore, the correlation between ZPI‐3 response to SDH activity in clinical tumor versus adjacent tissues suggests a framework for patient stratification. These findings introduce modification‐selective inhibition as a new paradigm for precise mitochondrial modulation and highlight the potential of exploiting post‐translational vulnerabilities for selective metabolic intervention in cancer.

## Author Contributions

**Ning Wang**: software, investigation. **Lulu Kong**: methodology, formal analysis. **Shaohua Wei**: conceptualization, funding acquisition, resources, project administration. **Kebin Ye**: methodology, data curation. **Yongming Deng**: conceptualization, funding acquisition, resources, writing – original draft. **Lin Zhou**: writing – review and editing, funding acquisition, resources, investigation, writing – original draft, conceptualization. **Haowen Liu**: data curation, investigation. **Xinlu Bao**: investigation, data curation. **Wei Wang**: funding acquisition, resources, supervision, conceptualization. **Kaikai Xu**: conceptualization, writing – original draft, data curation.

## Conflicts of Interest

The authors declare no conflicts of interest.

## Supporting information




**Supporting File**: advs77046‐sup‐0001‐SuppMat.docx.

## Data Availability

The data that support the findings of this study are available from the corresponding author upon reasonable request.

## References

[1] G. A. Bezawork , J. Rohlena , L. Dong , K. Pacak , and J. Neuzil , “Mitochondrial Complex II: At the Crossroads,” Trends in Biochemical Sciences 42, no. 4 (2017): 312–325, 10.1016/j.tibs.2017.01.003. PMC7441821 28185716

[2] J. Yu , Q. Yao , T. Hu , et al., “The Role of HINT_3_ in Myocardial Ischemia‐Reperfusion Injury in Male Mice: Mechanisms Involving SDHA and its Acetylation,” Advanced Science 12, no. 33 (2025): 03109, 10.1002/advs.202503109. PMC12412524 40755357

[3] R. Kugapreethan , S. N. Elahee Doomun , J. Sacharz , et al., “Complex II Assembly Drives Metabolic Adaptation to OXPHOS Dysfunction,” Science Advances 11 (2025): adr6012. 10.1126/sciadv.adr6012 PMC12356253 40815660

[4] H. Wang , B. Huwaimel , K. Verma , J. Miller , and T. M. Germain , “Synthesis and Antineoplastic Evaluation of Mitochondrial Complex II (Succinate Dehydrogenase) Inhibitors Derived From Atpenin A5,” Chemmedchem 12, no. 13 (2017): 1033–1044, 10.1002/cmdc.201700196. PMC5557372 28523727

[5] H. Qiao , Z. Yuan , L. Xing , B. X. He , L. Y. Ma , and Z. X. He , “Innovative Medicinal Chemistry Strategies for Improving Target Binding Kinetics in Drug Discovery,” Journal of Medicinal Chemistry 68, no. 21 (2025): 22116–22144, 10.1021/acs.jmedchem.5c02299. 41147376

[6] Q. Wang , T. Yang , S. Li , et al., “Unimolecular Self‐Assembled Hemicyanine–Oleic Acid Conjugate Acts as a Novel Succinate Dehydrogenase Inhibitor to Amplify Photodynamic Therapy and Eliminate Cancer Stem Cells,” Research 6 (2023): 0223, 10.34133/research.0223. PMC10482163 37680304

[7] K. Ma , H. Cheng , L. Wang , et al., “Succinate preserves CD^8+^ T cell fitness to augment antitumor immunity,” Immunity 58, no. 10 (2025): 2505–2523, 10.1016/j.immuni.2025.07.017. 40816269

[8] K. C. Mangalhara , S. K. Varanasi , M. A. Johnson , et al., “Manipulating Mitochondrial Electron Flow Enhances Tumor Immunogenicity,” Science 381, no. 6664 (2023): 1316–1323, 10.1126/science.abq1053. PMC11034774 37733872

[9] S. T. Li , D. Huang , S. Shen , et al., “Myc‐Mediated SDHA Acetylation Triggers Epigenetic Regulation of Gene Expression and Tumorigenesis,” Nature Metabolism 2, no. 3 (2020): 256–269, 10.1038/s42255-020-0179-8. 32694775

[10] Y. Liu , K. Liu , R. F. Thorne , et al., “Mitochondrial SENP2 Regulates the Assembly of SDH Complex Under Metabolic Stress,” Cell Reports 42, no. 2 (2023): 112041. 10.1016/j.celrep.2023.112041 36708515

[11] L. S. Huang , G. Sun , D. Cobessi , et al., “3‐Nitropropionic Acid Is a Suicide Inhibitor of Mitochondrial Respiration That, upon Oxidation by Complex II, Forms a Covalent Adduct With a Catalytic Base Arginine in the Active Site of the Enzyme,” Journal of Biological Chemistry 281, no. 9 (2006): 5965–5972, 10.1074/jbc.M511270200. PMC1482830 16371358

[12] L. L. Kong , K. K. Xu , X. L. Bao , et al., “mTORC1 Selective Nano‐Inhibitor by Disrupting the Lysosomal Arginine‐SLC38A9‐ mTORC1‐CDKs Axis for Precision Bladder Cancer Therapy,” Advanced Materials 37, no. 39 (2025): 2504798, 10.1002/adma.202504798. 40613244

[13] W. Wu , J. Huang , Y. Li , et al., “Endoplasmic Reticulum‐Targeting Iridium(III) Complexes Induce Pyroptosis and Enhance Immunogenic Cell Death in MDA‐MB‐231 Cells,” Journal of Medicinal Chemistry 68, no. 14 (2025): 14727–14739, 10.1021/acs.jmedchem.5c00883. 40639936

[14] X. Zhao , X. Wang , W. Zhang , et al., “A Ferroptosis‐Inducing Arsenene‐Iridium Nanoplatform for Synergistic Immunotherapy in Pancreatic Cancer,” Angewandte Chemie International Edition 63, no. 15 (2024): 202400829, 10.1002/anie.202400829. 38349715

[15] X. Xiong , K. B. Huang , Y. Wang , et al., “Target Profiling of an Iridium(III)‐Based Immunogenic Cell Death Inducer Unveils the Engagement of Unfolded Protein Response Regulator BiP,” Journal of the American Chemical Society 144, no. 23 (2022): 10407–10416, 10.1021/jacs.2c02435. 35658433

[16] L. Bai , Z. Deng , M. Xu , et al., “CETSA‐MS‐Based Target Profiling of Anti‐Aging Natural Compound Quercetin,” European Journal of Medicinal Chemistry 267 (2024): 116203, 10.1016/j.ejmech.2024.116203. 38342014

[17] Y. Tu , L. Tan , H. Tao , Y. Li , and H. Liu , “CETSA and Thermal Proteome Profiling Strategies for Target Identification and Drug Discovery of Natural Products,” Phytomedicine 116 (2023): 154862, 10.1016/j.phymed.2023.154862. 37216761

[18] Y. Lu , F. Y. Wang , M. S. Levine , et al., “Oxoisoaporphine Alkaloid Iridium(III) Derivative: An Immunogenic Cell Death Inducer That Engages the Autophagy‐Dependent Regulator Cathepsin D,” Journal of the American Chemical Society 147, no. 18 (2025): 15216–15228, 10.1021/jacs.5c00255. 40279467

[19] S. Jin , C. Lin , Y. Wang , et al., “Cannabidiol Analogue CIAC001 for the Treatment of Morphine‐Induced Addiction by Targeting PKM2,” Journal of Medicinal Chemistry 66, no. 16 (2023): 11498–11516, 10.1021/acs.jmedchem.3c01029. 37531582

[20] W. J. Wang , Y. Y. Ling , Y. Shi , et al., “Identification of Mitochondrial ATP Synthase as the Cellular target of Ru‐Polypyridyl‐ β ‐Carboline Complexes by Affinity‐Based Protein Profiling,” National Science Review 11, no. 8 (2024): nwae234, 10.1093/nsr/nwae234. PMC11304990 39114378

[21] S. Akabane , M. Uno , and N. Tani , “PKA Regulates PINK1 Stability and Parkin Recruitment to Damaged Mitochondria Through Phosphorylation of MIC60,” Molecular Cell 62, no. 3 (2016): 371–384, 10.1016/j.molcel.2016.03.037. 27153535

[22] M. A. Nengroo , A. T. Klein , H. S. Carr , et al., “Accumulation of Succinate Suppresses de novo Purine Synthesis Through Succinylation‐Mediated Control of the Mitochondrial Folate Cycle,” Molecular Cell 85, no. 22 (2025): 4215–4228, 10.1016/j.molcel.2025.10.002. PMC12616479 41161310

[23] M. Schuhmacher , M. S. Staege , A. Pajic , et al., “Control of Cell Growth by c‐Myc in the Absence of Cell Division,” Current Biology 9, no. 21 (1999): 1255–1258, 10.1016/S0960-9822(99)80507-7. 10556095

[24] Y. Liu , Z. Zhou , J. Hou , et al., “Tumor Selective Metabolic Reprogramming as a Prospective PD‐L1 Depression Strategy to Reactivate Immunotherapy,” Advanced Materials 34, no. 41 (2022): 2206121, 10.1002/adma.202206121. 36017886

[25] T. F. Slater , B. Sawyer , and U. Struli , “Studies on Succinate‐Tetrazolium Reductase Systems,” Biochimica et Biophysica Acta 77 (1963): 383–393, 10.1016/0006-3002(63)90513-4. 14089413

[26] R. Wei , Y. Chen , Q. Yang , et al., “Nanoenzyme‐Anchored Mitofactories Boost Mitochondrial Transplantation to Restore Locomotor Function After Paralysis Following Spinal Cord Injury,” ACS Nano 19, no. 4 (2025): 4403–4421, 10.1021/acsnano.4c12557. 39853984

[27] P. I. Tsai , C. H. Lin , and C. H. Hsieh , “PINK1 Phosphorylates MIC60/Mitofilin to Control Structural Plasticity of Mitochondrial Crista Junctions,” Molecular Cell 69, no. 5 (2018): 744–756, 10.1016/j.molcel.2018.01.026. 29456190

[28] A. Suomalainen and J. Nunnari , “Mitochondria at the Crossroads of Health and Disease,” Cell 187, no. 11 (2024): 2601–2627, 10.1016/j.cell.2024.04.037. 38788685

[29] W. J. Wang , Y. Y. Ling , Y. M. Zhong , Z. Y. Li , C. P. Tan , and Z. W. Mao , “Ferroptosis‐Enhanced Cancer Immunity by a Ferrocene‐Appended Iridium(III) Diphosphine Complex,” Angewandte Chemie International Edition 61, no. 16 (2022): 202115247, 10.1002/anie.202115247. 34965011

[30] H. Gu , P. Yuan , J. Zhang , et al., “Tuning Exciton Coupling of Non‐Conjugated Cyanine Dimers for Efficient Photodynamic Immunotherapy,” Journal of the American Chemical Society 147, no. 24 (2025): 20778–20789, 10.1021/jacs.5c04044. 40471127

[31] C. Simó , M. Serra Casablancas , A. C. Hortelao , et al., “Urease‐Powered Nanobots for Radionuclide Bladder Cancer Therapy,” Nature Nanotechnology 19 (2024): 554–564. 10.1038/s41565-023-01577-y PMC11026160 38225356

[32] Y. Zhang , F. Huo , Q. Cao , et al., “FimH Confers Mannose‐Targeting Ability to Bacillus Calmette‐Guerin for Improved Immunotherapy in Bladder Cancer,” Journal for ImmunoTherapy of Cancer 10, no. 3 (2022): 003939, 10.1136/jitc-2021-003939. PMC8971803 35361729

[33] J. Sun , R. Chu , X. Wu , et al., “Anti‐Biopassivated Reticular Micromotors for Bladder Cancer Therapy,” Journal of the American Chemical Society 147, no. 21 (2025): 17936–17945, 10.1021/jacs.5c02949. 40378338

[34] M. Y. Lucero and J. Chan , “Photoacoustic Imaging of Elevated Glutathione in Models of Lung Cancer for Companion Diagnostic Applications,” Nature Chemistry 13 (2021): 1248–1256. 10.1038/s41557-021-00804-0 PMC8629919 34697400

[35] A. M. Kamat , N. M. Hahn , J. A. Efstathiou , et al., “Bladder Cancer,” The Lancet 388, no. 10061 (2016): 2796–2810, 10.1016/S0140-6736(16)30512-8. 27345655

[36] M. Leyderman , T. Chandrasekar , P. Grivas , et al., “Metastasis Development in Non‐Muscle‐Invasive Bladder Cancer,” Nature Reviews Urology 22, no. 6 (2025): 375–386, 10.1038/s41585-024-00963-y. 39567681

[37] X. Wang , X. Zhang , K. Cao , et al., “Cardiac Disruption of SDHAF4‐Mediated Mitochondrial Complex II Assembly Promotes Dilated Cardiomyopathy,” Nature Communications 13, no. 1 (2022): 3947, 10.1038/s41467-022-31548-1. PMC9270418 35803927

