# Supplementary material for: Modification‐Selective Inhibition of Succinate Dehydrogenase a Enables Tumor‐Restricted Mitochondrial Modulation
Source: Adv Sci (Weinh). 2026 Aug 19;13(62):e77046. doi: 10.1002/advs.77046 (PMC13487864; doi:10.1002/advs.77046)
Supplement: Supplementary file 1 [file ADVS-13-e77046-s001.docx]

**Supporting information**

**Modification-Selective Inhibition of Succinate Dehydrogenase A Enables Tumor-Restricted Mitochondrial Modulation**

Kaikai Xu,^a +^ Lulu Kong,^a +^ Kebin Ye,^a+^ Xinlu Bao,^a^ Haowen Liu, ^a^ Ning Wang,^d^ Yongming Deng,^c^ * Wei Wang,^b^ * Shaohua Wei,^a^* Lin Zhou ^a^ *

^a^ College of Chemistry and Materials Science, Jiangsu Key Laboratory of Bio-functional Materials, Jiangsu Collaborative Innovation Centre of Biomedical Functional Materials, Nanjing Normal University, Nanjing, 210023, China.

^b^ School of Chemistry and Chemical Engineering, Yancheng Institute of Technology, Yancheng, 224051, China.

^c^ Department of Urology, Nanjing Drum Tower Hospital, the Affiliated Hospital of Nanjing University Medical School, Institute of Urology, Nanjing University, Nanjing, 210008, China.

^d^ School of Science, Key Laboratory of High Performance Scientific Computation, Xihua University, Chengdu 610039, China.

^+^ These authors contributed equally to the work

* Corresponding authors

# 1 Materials and Methods

## 1.1 Materials and Reagents

4-Nitrophthalonitrile, p-hydroxybenzoic acid, zinc acetate, and (3AS,4S,6AR)-N-[2-[2-(2-azidoethoxy)ethoxy]ethyl]hexahydro-2-oxo-1H-thieno[3,4-D]imidazole-4-pentanamide (Biotin PEG3 azide) were purchased from Aladdin Chemicals Co., Ltd. (China). *N,N*-diisopropylethylamine (DIPEA) and propargyl bromide were obtained from Macklin Biochemical Co., Ltd. (China). 4-(Dimethylamino)pyridine (DMAP) was purchased from J&K Scientific Ltd. (China). 1,10-Phenanthroline-5,6-dione, 4-(Boc-amino)benzaldehyde, 2-phenylpyridine, iridium(III) chloride hydrate, 2-(3-bromophenyl)pyridine, 3-amino-4-hydroxybenzoic acid, di-tert-butyl dicarbonate, propargylamine, tris[(1-benzyl-1H-1,2,3-triazol-4-yl)methyl]amine (TBTA), tris(2-carboxyethyl)phosphine hydrochloride (TCEP), anhydrous copper(II) sulfate, potassium acetate, tris(dibenzylideneacetone)dipalladium(0) [Pd₂(dba)₃], 1-[bis(dimethylamino)methylene]-1H-benzotriazolium 3-oxide hexafluoroborate (TBTU), 2-(7-aza-1H-benzotriazol-1-yl)-*N,N,N′,N′*-tetramethyluronium hexafluorophosphate (HATU), and 2-(2-methoxy-4-nitrophenyl)-3-(4-nitrophenyl)-5-(2,4-disulfophenyl)-2H-tetrazolium monosodium salt (WST-8) were purchased from Bide Pharmatech Co., Ltd. (China). Triethylene glycol and trimethylsilyldiazomethane (TMSCHN₂, 2 M in hexane) were obtained from Energy Chemical (China). 1,8-diazabicyclo[5.4.0]undec-7-ene (DBU), 9,10-diphenylanthracene propionic acid (ADPA), and 2′,7′-dichlorodihydrofluorescein diacetate (DCFH-DA) were purchased from Sigma-Aldrich (Shanghai, China). Ethylene glycol monomethyl ether, dichloromethane, methanol, ethanol, n-pentanol, *N,N*-dimethylformamide (DMF), sodium hydroxide, and anhydrous dimethyl sulfoxide (DMSO) were obtained from Sinopharm Chemical Reagent Co., Ltd. (China).

RPMI-1640 medium was purchased from Jiangsu Keygen Biotech Corp., Ltd. (China). Dulbecco’s modified Eagle medium (DMEM) and F-12K medium were purchased from Gibco (USA). Fetal bovine serum (FBS) was obtained from Sijiqing Co., Ltd. (China). 3-[4,5-dimethylthiazol-2-yl]-2,5-diphenyltetrazolium bromide (MTT) and the mitochondrial probe MitoTracker Green were obtained from Thermo Fisher Scientific. The BeyoClick™ EdU-488 Cell Proliferation Kit, Neutral Red Cell Proliferation and Cytotoxicity Assay Kit, Fast Silver Stain Kit, BeyoMag™ Streptavidin Magnetic Beads, Immunoprecipitation Kit (Protein A+G Magnetic Beads), protease inhibitor cocktail, Mitochondrial Superoxide Assay Kit with MitoSO™ Red and deacetylase inhibitor mixture (40 μM trichostatin A, 1 mM EX-527, 400 mM nicotinamide, and 200 mM sodium butyrate in 70% DMSO) were purchased from Beyotime Biotechnology (Shanghai, China). Gel filtration chromatography filler (200-400 mesh) was purchased from Bio-Rad (USA). The succinate dehydrogenase (SDH) activity assay kit was obtained from Solarbio Science & Technology Co., Ltd. (Beijing, China), and the succinate (SUCN) ELISA kit was purchased from Shanghai Hengyuan Biotechnology Co., Ltd. (China).

Primary antibodies against SDHA (Proteintech, 66588-1-Ig), HSPA9 (Proteintech, 14887-1-AP), AIFM1 (Proteintech, 17984-1-AP), NDUFS (Proteintech, 124444-1-AP), ACADVL (Proteintech, 14527-1-AP), and pan-acetylation (Proteintech, 662589-1-AP) were purchased from Wuhan Proteintech Group, Inc. (China). The MIC_60_ antibody (Abcam, ab137057) was obtained from Abcam (Shanghai) Trading Co., Ltd. The recombinant human-derived SDHA protein was purchased from MedChemExpress (MCE, HY-P704801).

## 1.2 Instruments and Characterization

^1^H and ^13^C nuclear magnetic resonance (NMR) spectra were recorded on a Bruker Avance 400 MHz NMR spectrometer (Germany). Matrix-assisted laser desorption/ionization time-of-flight mass spectrometry (MALDI-TOF MS) was performed on a Bruker UltrafleXtreme spectrometer (Germany). Fluorescence (FL) spectra were recorded on a PerkinElmer LS-50B fluorescence spectrophotometer. Ultraviolet-visible (UV-Vis) absorption spectra were obtained using a Yuanxi UV-6100 spectrophotometer (Shanghai, China). Cellular fluorescence images were captured using Nikon ECLIPSE Ti-s and ECLIPSE Ji inverted fluorescence microscopes (Japan).

Cell viability was measured using a MULTISKAN FC microplate reader (Thermo Fisher Scientific, USA). Cellular reactive oxygen species (ROS) levels were analyzed by flow cytometry (XL MCL, ESCALAB, China). Western blot images were acquired using a Tanon-5200 E-Gel imaging system (China). In vivo fluorescence imaging of small animals was conducted using a PerkinElmer optical imaging system (USA). Real-time monitoring of cell proliferation was performed with a CM100-α real-time cell analysis system (Liucandou Medical Technology Co., Ltd., Shanghai, China), which detects impedance signals on microelectrode-coated plates to assess cell growth dynamics. Biochemical parameters in animal serum were analyzed using a Mindray BS-350S automatic biochemical analyzer (Shenzhen, China). Electrochemical measurements were performed on a CHI660E electrochemical workstation (Chenhua, Shanghai, China). Transmission electron microscopy (TEM) images were obtained using a Hitachi H-7650 TEM (Japan). Microscale Thermophoresis (MST) measurements were performed using a Monolith NT.115 Pico instrument (NanoTemper Technologies, Germany). Serum biochemical parameters in mice were measured using the Mindray fully automated veterinary biochemistry analyzer BS-240VET.

T24, SV-HUC-1, 5637 and UM-UC-3 cell lines were obtained from the Cell Bank of the Chinese Academy of Sciences and authenticated by short tandem repeat (STR) profiling. MB49 and 5637 cell lines were kindly provided by Nanjing Drum Tower Hospital, Medical School of Nanjing University, and also authenticated by STR analysis.

Female C57BL/6 mice (20 g) were purchased from Hangzhou Medical College. All animal experiments were performed in accordance with the guidelines approved by the Animal Ethics Committee of Nanjing Normal University (approval number: IACUC-202309016). This study was conducted in accordance with the ethical principles of the World Medical Association Declaration of Helsinki. All procedures involving human samples were approved by the Ethics Committee of Nanjing Drum Tower Hospital, Medical School of Nanjing University (approval number: 2024-298-1). Informed consent was obtained from all individual participants included in the study. Patient Consent for Publication: no written consent has been obtained from the patients as there is no patient identifiable data included in this case report/series.

## 1.3 Chemical Synthesis

**1.3.1 Synthesis of Mono-Substituted Carboxyphthalocyanine (ZnPc-COOH, Scheme 1)**

**(1) Synthesis of compound A1.**

Compound A1 was synthesized according to a previously reported procedure. In a 100 mL three-necked round-bottom flask, 4-nitrophthalonitrile (5.0 g, 28.88 mmol), p-hydroxybenzoic acid (4.79 g, 34.66 mmol), K₂CO₃ (5.99 g, 43.34 mmol), and DMF (10 mL) were added successively. The reaction mixture was stirred at 75 °C for 8 h under a nitrogen atmosphere. Upon completion, the mixture was cooled to room temperature and poured into 50 mL of ice water. The pH of the suspension was adjusted to 2–3 with concentrated HCl (12 M), resulting in the formation of a yellow precipitate. The solid was collected by filtration, washed with distilled water until neutral, and dried under vacuum to afford a yellow solid (5.63 g, yield 73.78%). ^1^H NMR (400 MHz, CDCl_3_): δ (ppm) 8.16 (d, *J* = 8.8 Hz, 1H), 8.03 (d, *J* = 8.8 Hz, 2H), 7.94 (d, *J* = 8 Hz, 1H), 7.54 (dd, *J* = 8.8, 2.4 Hz, 1H), 7.27 (d, *J* = 8.8 Hz, 1H). ^13^C NMR (100 MHz, CDCl_3_): d (ppm) 167.1, 160.3, 158.1, 136.9, 132.4, 128.7, 124.2, 123.7, 120.0, 117.3, 116.3, 115.8, 109.6.

**Scheme S1.** The synthesis route for ZnPc-COOH.

**(2) Synthesis of ZnPc-COOH**

In a 100 mL three-necked round-bottom flask, compound A1 (0.35 g, 1.32 mmol), o-phthalonitrile (1.69 g, 13.2 mmol), anhydrous zinc acetate (1.24 g, 5.68 mmol), and n-pentanol (25 mL) were added sequentially. The reaction mixture was stirred under a nitrogen atmosphere and heated to 100 °C for 30 min. Subsequently, DBU (0.85 mL) was added dropwise, and the mixture was further heated at 140 °C for 12 h. During the reaction, the color of the solution gradually changed from brownish to dark green. After cooling to room temperature, the solvent was removed under reduced pressure. The resulting crude dark-green solid was dispersed in a mixture of methanol/concentrated HCl (v/v = 40 mL:2 mL) and refluxed at 80 °C for 3 h. After cooling, the precipitate was collected by filtration and further refluxed in fresh methanol/concentrated HCl solution (v/v = 40:2) until the supernatant became colorless. The solid was filtered, washed with deionized water, and dried under vacuum to give 1.5 g of a dark-green crude product, which contained both unsubstituted and mono-substituted zinc phthalocyanines.

The obtained crude product (0.75 g) was dissolved in DMF (30 mL) in a 100 mL round-bottom flask, followed by the addition of triethylene glycol (1.20 g, 6.3 mmol), TBTU (1.32 g, 4.20 mmol), and DMAP (0.51 g, 4.20 mmol). The reaction mixture was stirred at room temperature for 12 h. After completion, the solution was poured into 300 mL of water to precipitate a large amount of dark-green solid. The crude product was collected, dried, and subjected to silica gel column chromatography (DCM/MeOH = 40:1, v/v) to obtain a green intermediate. Further purification by size-exclusion chromatography using Bio-Beads S-X3 and DMF as the eluent afforded a green solid (72 mg, yield 12.3%). ^1^H NMR (400 MHz, DMSO-*d_6_*): δ (ppm) 9.32 - 8.73 (m, 8H), 8.56 (s, 1H), 8.29 - 8.16 (m, 7H), 7.78 (s, 1H), 7.57 (d, *J* = 8.8 Hz, 2H), 4.47 (t, *J* = 4.0 Hz, 2H), 3.81 (t, *J* = 4.8 Hz, 2H), 3.65-3.62 (m, 4H), 3.43-3.39 (m, 4H). MS (MALDI-TOF, [M + H]^+^) m/z: calcd for C_47_H_36_N_8_O_7_Zn: 888.20. Found: [M + H]^+^ 888.01.

The obtained dark-green solid (from the previous step) was dissolved in THF (2 mL), followed by the slow addition of methanol (8 mL) and 10% aqueous NaOH solution (3 mL). The mixture was heated at 100 °C for 10 h. After cooling to room temperature, the pH was adjusted to 2 using 1 M HCl, resulting in a dark-green precipitate. The solid was collected by filtration, washed with deionized water until the filtrate reached approximately pH 6, and dried under vacuum to yield 65 mg of a dark-green solid. ^1^H NMR (400 MHz, DMSO-*d_6_*): δ (ppm) 9.36 - 9.23 (m, 6H), 9.11 (d, *J* = 6.8 Hz, 1H), 8.73 (s, 1H ), 8.28 - 8.17 (m, 8H), 7.91 (d, *J* = 8.4 Hz, 1H), 7.57 - 7.44 (m, 2H). MS (MALDI-TOF，[M + H]^+^) m/z: calcd for C_39_H_20_N_8_O_3_Zn: 712.09. Found: [M + H]^+^ 712.85.

**1.3.2 Synthesis of Ir–NH₂ (Scheme 2)**

**Scheme S2.** The synthesis route for Ir-NH_2_.

**(1) Synthesis of Compound B1**

In a 50 mL round-bottom flask, 1,10-phenanthroline-5,6-dione (210 mg, 1 mmol), 4-(Boc-amino) benzaldehyde (232 mg, 1.1 mmol), ammonium acetate (1.92 g, 25 mmol), and glacial acetic acid (10 mL) were added sequentially. The mixture was heated to 85 °C under a nitrogen atmosphere and stirred for 6 h. After completion, the reaction mixture was cooled to room temperature and concentrated under reduced pressure to remove the solvent. The residue was purified by silica gel column chromatography using dichloromethane/methanol (v/v = 10:1) as the eluent to yield compound B1 as a yellow solid (250 mg, 61.0% yield). ^1^H NMR (400 MHz, DMSO-*d_6_*): δ (ppm) 9.68 (s, 1H), 9.02 (d, *J* = 4.4 Hz, 2H), 8.94 (d, *J* = 8.4 Hz, 2H), 8.19 (d, *J* = 8.4 Hz, 2H), 7.83 (dd, *J* = 8.4, 4.4 Hz, 2H), 7.68 (d, *J* = 8.4 Hz, 2H), 1.56 (s, 9H). ^13^C NMR (100 MHz, DMSO-*d_6_*): d (ppm) 153.2, 151.2, 148.1, 143.9, 141.4, 130.0, 127.4, 124.6, 123.7, 118.6, 79.9, 49.1, 28.5.

**(2) Synthesis of Compound B2**

Compound B2 was synthesized according to a literature procedure. In a 100 mL round-bottom flask, 2-phenylpyridine (96.15 mg, 0.62 mmol) and iridium(III) chloride hydrate (100 mg, 0.31 mmol) were dissolved in 2-methoxyethanol (30 mL) and H₂O (10 mL). The mixture was heated to 110 °C under nitrogen and stirred for 24 h. After cooling to room temperature, the reaction mixture was poured into 50 mL of ice water to afford a yellow precipitate, which was collected by filtration and washed with water and methanol until the filtrate was colorless. The product B2 was obtained as a yellow solid (72 mg, 61.0% yield). ^1^H NMR(400 MHz，DMSO-*d_6_*): δ (ppm) 9.81 (d, *J* = 5.6 Hz, 3H), 9.54 (d, *J* = 5.2 Hz, 2H), 8.26 (d, *J* = 7.6 Hz, 2H), 8.18 (d, *J* = 8.0 Hz, 1H), 8.10 (t, *J* = 8.4 Hz, 2H), 8.02 (t, *J* = 8.4 Hz, 2H), 7.79 (d, *J* = 7.6 Hz, 2H), 7.74 (d, *J* = 7.6 Hz, 2H)，7.58 (t, *J* = 6.0 Hz, 2H), 7.46 (t, *J* = 6.8 Hz, 2H), 6.90 (t, *J* = 7.2 Hz, 2H), 6.84 (t, *J* = 7.6 Hz, 2H), 6.77 (t, *J* = 7.2 Hz, 2H), 6.69 (t, *J* = 7.6 Hz, 2H), 6.25 (d, *J* = 7.6 Hz, 2H), 5.66 (d, *J* = 7.6 Hz, 2H). ^13^C NMR (100 MHz, d_6_-DMSO): d (ppm) 168.5, 162.4, 151.7, 145.3, 136.1, 130.6, 129.1, 123.6, 122.1, 121.3, 118.4, 102.8.

**(3) Synthesis of Compound B3**

In a 50 mL round-bottom flask, dichloro[2-(2-pyridyl)phenyl]iridium(III) dimer (B2, 200 mg, 0.19 mmol) and tert-butyl (4-(1H-imidazo[4,5-f][1,10]phenanthrolin-2-yl)phenyl)carbamate (B1, 156 mg, 0.38 mmol) were dissolved in dichloromethane/methanol (10 mL/20 mL). The reaction mixture was stirred at 55 °C for 12 h. After solvent removal under reduced pressure, the residue was purified by silica gel chromatography using dichloromethane/methanol (v/v = 10:1) as the eluent to afford compound B3 as a yellow powder (267 mg, 77.16% yield). ^1^H NMR (400 MHz，DMSO-*d_6_*): δ (ppm) 9.74 (s, 1H), 9.40 (d, *J* = 8.0 Hz, 1H), 9.18 (d, *J* = 8.0 Hz, 1H), 8.28 (t, *J* = 8.8 Hz, 4H), 8.22 - 8.03 (m, 4H), 7.96 (d, *J* = 8.0 Hz, 2H), 7.88 (t, *J* = 7.6 Hz, 2H), 7.71 (d, *J* = 8.8 Hz, 2H), 7.51 (d, *J* = 5.2 Hz, 2H), 7.07 (t, *J* = 7.6 Hz, 2H), 7.04 - 6.92 (m, 4H), 6.30 (d, *J* = 7.2 Hz, 2H), 1.52 (s, 9H). ^13^C NMR (100 MHz, d_6_-DMSO): d (ppm) 139.1, 131.7, 130.7, 128.0, 125.5, 124.3, 123.5, 122. 8, 120.4, 118.6, 99.9, 80.0, 49.1, 28.6. MS (MALDI-TOF, [M - Cl]^+^) m/z: calcd for C_46_H_37_IrN_7_O_2_: 912.26. Found: [M - Cl]^+^ 911.62.

**(4) Synthesis of Ir–NH₂**

Compound B3 (130 mg, 0.14 mmol) was dissolved in ethanol/H₂O (7 mL/2 mL), followed by the addition of concentrated HCl (2 mL). The mixture was refluxed at 100 °C for 6 h in a sealed tube. After cooling to room temperature, ethanol was removed under reduced pressure, and the residue was neutralized to pH 10–12 with 5% NaOH aqueous solution. The resulting reddish-brown precipitate was collected by centrifugation, washed three times with water, and dried under vacuum to yield compound Ir–NH₂ as a red-brown solid (100 mg, 88.49% yield). ^1^H NMR (400 MHz, DMSO-*d_6_*): δ (ppm) 9.03 (d, *J* = 6.8 Hz, 2H), 8.26 (d, *J* = 8.0 Hz, 2H), 8.06 (d, *J* = 8.4 Hz, 2H), 7.95 (d, *J* = 7.2 Hz, 4H), 7.80 - 7.68 (m, 4H), 7.47 (d, *J* = 5.6 Hz, 2H), 7.25 - 6.80 (m, 6H), 6.65 (d, *J* = 8.0 Hz, 2H), 6.31 (d, *J* = 7.6 Hz, 2H). MS (MALDI-TOF, [M - Cl]^+^) m/z: calcd for C_41_H_29_IrN_7_: 812.21. Found: [M - Cl]^+^ 812.94.

**1.3.3 Synthesis of ZPI-1 (Scheme 3)**

**Scheme S3.** The synthesis route for ZPI-1.

A 50 mL round-bottom flask was charged with compound A (40 mg, 0.056 mmol), TBTU (17.9 mg, 0.056 mmol), DMAP (5.8 mg, 0.056 mmol), and DMF (6 mL). The mixture was stirred in an ice-water bath for 1 h. Then, compound B4 (54 mg, 0.067 mmol) dissolved in 2 mL of dichloromethane (DCM) was added dropwise to the reaction mixture, and the reaction was allowed to proceed at room temperature for 12 h. Upon completion, DCM was removed under reduced pressure, and 50 mL of distilled water was added to the mixture, resulting in the formation of a yellowish-green precipitate. The solid was collected by centrifugation, washed three times each with distilled water and methanol, and dried under vacuum to afford the crude product. The crude material was further purified by gel permeation chromatography on Bio-Beads S-X3 using DMF as the eluent. The first greenish-yellow fraction was collected to yield compound ZPI-1 as a dark green solid (12.3 mg, 14.56% yield). ^1^H NMR (400 MHz, DMSO-*d_6_*):,δ (ppm) 9.26 - 9.20 (m, 11H), 9.20 - 9.0 (m, 6H), 8.64 (s, 1H), 8.27 (d, *J* = 8 Hz, 2H), 8.20 - 8.10 (m, 10H), 8.10 - 8.00 (m, 1H), 7.99 (t, *J* = 8 Hz, 3H), 7.56 (t, *J* = 8 Hz, 3H), 7.70 - 7.60 (m, 1H), 7.60- 7.41 (m, 4H), 7.05 (t, *J* = 8 Hz, 3H), 7.03 - 6.90 (m, 3H), 6.30 (d, *J* = 8 Hz, 2H), 5.78 - 5.69 (m, 2H). MS (MALDI-TOF, [M - Cl]^+^) m/z: calcd for C_80_H_47_IrN_15_O_2_Zn: 1506.97. Found: [M - Cl]^+^ 1506.80.

**1.3.4 Synthesis of ZPI-2 (Scheme 4)**

**(1) Synthesis of C2**

3-Amino-4-hydroxybenzoic acid (851 mg, 5.56 mmol), (Boc)₂O (1.27 g, 5.82 mmol), and DMF (7 mL) were added sequentially into a 100 mL round-bottom flask and heated at 70 °C for 2 h. After completion, the reaction mixture was cooled to room temperature and poured into 30 mL of water to precipitate a brown solid, which was collected by filtration and dried under vacuum to afford compound C1 (1.1 g) as a brownish-gray solid. The crude product was used directly for the next step without further purification.

Compound C1 (253 mg, 1 mmol) was dissolved in DCM (2 mL) and MeOH (6 mL) under nitrogen at 0 °C. TMSCHN₂ (2 mmol, 1 mL) was added dropwise and the reaction was stirred for 1 h in an ice bath, then at room temperature for 2 h. After quenching with two drops of acetic acid, the mixture was purified by silica gel chromatography (DCM/MeOH, v/v = 50:1) to yield compound C2 (234 mg, 87.64%) as an off-white solid. ^1^H NMR (400 MHz，DMSO-*d_6_*): δ (ppm) 10.8 (s, 1H), 8.31 (s, 1H), 7.93 (s, 1H), 7.54 (dd, *J* = 8.4, 2 Hz, 1H), 6.90 (d, *J* = 8.4 Hz, 1H), 3.70 (s, 3H), 1.47 (s, 9H).

**Scheme S4.** The synthesis route for ZPI-2.

**(2) Synthesis of C3**

4-Nitrophthalonitrile (259 mg, 1.5 mmol), compound C2 (267 mg, 1 mmol), and K₂CO₃ (487 mg, 1.5 mmol) were dissolved in DMF (10 mL) and stirred at 50 °C under nitrogen for 1 h. The mixture was poured into 30 mL of water, followed by addition of 5 mL saturated NaHCO₃ solution, and extracted with ethyl acetate. The crude product was purified by silica gel chromatography (petroleum ether/ethyl acetate, v/v = 2:1) to yield compound C3 (305 mg, 77.61%) as an off-white solid. ^1^H NMR (400 MHz, CDCl_3_): δ (ppm) 8.02 (dd, *J* = 8.4, 2 Hz, 1H), 7.78 (dd, *J* = 8.4, 2 Hz, 2H), 7.69 (d, *J* = 8.8 Hz, 1H), 7.62 (dd, *J* = 8.4, 2 Hz, 1H), 7.06 (d, *J* = 8.8 Hz, 1H),, 3.91 (s, 3H), 1.45 (s, 9H).

**(3) Synthesis of C5**

Compound C3 (300 mg, 0.76 mmol), phthalonitrile (1.07 g, 8.36 mmol), Zn(OAc)₂ (556 mg, 3.04 mmol), and n-pentanol (10 mL) were placed in a 100 mL three-necked flask and heated to 95 °C under nitrogen for 30 min. DBU (1 mL) was then added, and the reaction was continued at 140 °C for 12 h until the solution turned dark green. After solvent removal, the residue was chromatographed on silica gel (DCM/ethyl acetate, v/v = 4:1) to give a crude product, which was further purified by size-exclusion chromatography on Bio-Beads S-X3 (DMF eluent) to yield compound C4 as a dark green solid.

Compound C4 (50 mg, 0.06 mmol) was treated with TFA (2 mL) in DCM (10 mL) at room temperature for 3 h. The solvent was evaporated and the residue was precipitated with ether to afford compound C5 (106 mg, 17.52%) as a dark green solid. ^1^H NMR (400 MHz, DMSO-*d_6_*): δ (ppm) 9.45 - 8.97 (m, 6H), 8.61 (s, 1H), 8.36 - 8.04 (m, 5H), 7.81 (s, 1H), 8.36 - 8.04 (m, 5H), 7.81 (d, *J* = 8.4 Hz, 2H), 7.40 (m, 2H), 6.85 (d, *J* = 8.4 Hz, 2H), 4.21 (t, *J* = 6.8 Hz, 2H), 1.76 - 7.57 (m, 2H), 1.46 - 1.02 (m, 4H), 0.93 (t, *J* = 7.6 Hz, 3H). MS (MALDI-TOF, [M + H]^+^) m/z: calcd for C_44_H_31_N_9_O_3_Zn: 797.18. Found: [M + H]^+^ 799.73.

**(4) Synthesis of C7**

Compound C5 (30 mg, 0.04 mmol) was dissolved in DMSO (3 mL) and heated to 80 °C for 30 min, followed by the addition of propargyl bromide (50 μL). The reaction continued for 8 h, then poured into 10 mL water and extracted with ethyl acetate. The crude product was purified by silica gel chromatography (DCM/MeOH, v/v = 20:1) to afford compound C6 (20 mg).

Compound C6 (20 mg, 0.02 mmol) was then hydrolyzed with LiOH (5 mg/mL in water, 1.5 mL) in THF (2 mL) and MeOH (4 mL) under reflux at 80 °C for 12 h. After acidification (1 M HCl, pH = 3–4), a dark green solid precipitated and was collected to yield compound C6 (15 mg, 85.25%). ^1^H NMR (400 MHz, DMSO-*d_6_*): δ (ppm) 9.45 - 9.22 (m, 6H), 8.71 (s, 1H), 8.34 - 8.17 (m, 4H), 8.16 - 8.05 (m, 1H), 7.95 (s, 1H), 7.78 - 7.62 (m, 1H), 7.18 - 7.03 (m, 2H), 6.65 - 6.57 (m, 2H), 3.84 (dd, *J* = 6.4, 2.4 Hz, 2H), 3.05 (d, *J* = 2.0 Hz, 1H). MS (MALDI-TOF, [M + H]^+^) m/z: calcd for C_42_H_23_N_9_O_3_Zn: 765.12. Found: [M + H]^+^ 766.64.

**(5) Synthesis of ZPI-2**

Compound C6 (31.75 mg, 0.042 mmol), compound B3 (45.93 mg, 0.05 mmol), HATU (80 mg, 0.21 mmol), triethylamine (21 mg, 0.21 mmol), and DMAP (5 mg, 0.042 mmol) were dissolved in DMF (5 mL) and stirred at room temperature for 48 h. Additional portions of HATU (40 mg, 0.105 mmol) and DMAP (2.5 mg, 0.021 mmol) were added every 6 h during the reaction. After completion, the mixture was poured into 50 mL of water to precipitate a blue-green solid, which was collected and purified twice by silica gel chromatography (DCM/MeOH, v/v = 30:1). The crude product was refluxed in methanol (20 mL) with concentrated HCl (3 mL) at 80 °C for 6 h, followed by neutralization with saturated NaHCO₃ solution. The resulting blue solid was filtered, washed (H₂O ×3, acetone ×3), and dried under vacuum to afford ZPI-2 (9 mg, 13.84%). ^1^H NMR (400 MHz, DMSO-*d_6_*): δ (ppm) 9.37 (d, *J* = 7.6 Hz, 2H), 9.32 - 9.16 (m, 5H), 9.09 (d, *J* = 6.4 Hz, 1H), 8.72 (s, 1H), 8.26 (t, *J* = 7.6 Hz, 4H), 8.28 - 8.08 (m, 8H), 8.03 (dd, *J* = 8.4, 5.2 Hz, 2H), 7.96 (d, *J* = 7.6 Hz, 2H), 7.92 - 7.80 (m, 3H), 7.57- 7.48 (m, 3H), 7.14 (t, *J* = 7.6 Hz, 2H), 7.07 (t, *J* = 7.6 Hz, 2H), 7.02 - 6.89 (m, 5H), 6.78 - 6.58 (m, 2H), 6.30 (d, *J* = 7.6 Hz, 2H), 3.89 (d, *J* = 2.4 Hz, 2H), 3.10 (t, *J* = 2.4 Hz, 1H). MS (MALDI-TOF, [M - Cl]^+^) m/z: calcd for C_83_H_50_IrN_16_O_2_Zn: 1559.32. Found: [M - Cl]^+^ 1560.88.

**1.3.5 Synthesis of ZPI-3** **(Scheme 5)**

**Scheme S5.** The synthesis route for ZPI-3.

A 50 mL round-bottom flask was charged sequentially with compound ZnPc-COOH (30 mg, 0.042 mmol), compound B3 (45.93 mg, 0.05 mmol), HATU (80 mg, 0.21 mmol), triethylamine (21 mg, 0.21 mmol), DMAP (5 mg, 0.042 mmol), and DMF (5 mL). The reaction mixture was stirred at room temperature for 72 h. Additional portions of HATU (40 mg, 0.105 mmol) and DMAP (2.5 mg, 0.021 mmol) were added every 12 h during the reaction. Upon completion, the mixture was poured into distilled water (50 mL) to precipitate a blue-green solid. The solid was collected by centrifugation, blended with silica gel, and purified by column chromatography using DCM/MeOH (v/v = 30:1) as the eluent to afford a crude product. The crude material was dissolved in MeOH (30 mL), followed by addition of concentrated HCl (3 mL), and refluxed at 80 °C for 6 h. After the reaction was complete, the solvent was removed under reduced pressure. The residue was treated with distilled water (20 mL) and saturated NaHCO₃ solution (5 mL), producing a blue solid. The precipitate was collected by centrifugation, washed three times each with distilled water and acetone, and dried under vacuum to afford ZPI-3 as a blue-green solid (10.7 mg, 16.89% yield). ^1^H NMR (400 MHz, DMSO-*d_6_*): δ (ppm) 9.54 - 9.19 (m, 11H), 8.24 (s, 9H), 8.20 - 8.13 (m, 4H), 8.08 (s, 3H), 8.03 - 7.82 (m, 7H), 7.56 - 7.41 (m, 2H), 7.35 (d, *J* = 8.0 Hz, 1H), 7.09 - 6.82 (m, 5H), 6.72 (d, *J* = 8.0 Hz, 1H), 6.29 (d, *J* = 8.0 Hz, 1H), 5.74 (s, 1H). MS (MALDI-TOF, [M - Cl]^+^) m/z: calcd for C_80_H_47_IrN_15_O_2_Zn: 1506.30. Found: [M - Cl]^+^ 1506.80.

**1.3.6 Synthesis of ZPI-4 (Scheme 6)**

**Scheme S6.** The synthesis route for ZPI-4.

A 25 mL round-bottom flask was charged with ZPI-3 (10 mg, 6.63 μmol) and methanol (6 mL) under a nitrogen atmosphere. The mixture was heated to 90 °C, and an excess of methyl iodide (0.2 mL) was added. The reaction was maintained for 24 h at this temperature. After completion, the mixture was cooled to room temperature and poured into water (20 mL), resulting in the precipitation of a dark green solid. The solid was collected by filtration, washed with water until the filtrate became colorless, and dried under vacuum to yield ZPI-4 as a dark green solid (5 mg, 48.61% yield). ^1^H NMR (400 MHz, DMSO-*d_6_*): δ (ppm) 9.52 - 9.22 (m, 7H), 9.12 (s, 1H), 8.84 (s, 1H), 8.54 - 8.32 (m, 3H), 8.32 - 8.18 (m, 6H), 8.05 (dd, *J* = 8.4, 5.2 Hz, 2H), 7.96 (d, *J* = 8.0 Hz, 2H), 7.88 (t, *J* = 7.6 Hz, 2H), 7.51 (d, *J* = 6.0 Hz, 2H), 7.44 (d, *J* = 6.0 Hz, 1H), 7.07 (t, *J* = 7.2 Hz, 2H), 7.04 - 6.93 (m, 4H), 6.87 (d, *J* = 8.0 Hz, 1H), 6.72 (d, *J* = 8.0 Hz, 1H), 6.30 (d, *J* = 7.2 Hz, 2H), 3.17 (s, 9H). MS (MALDI-TOF, [M - 2I]^+^) m/z: calcd for C_83_H_54_IrN_15_O_2_Zn: 1549.35. Found: [M - 2I]^+^ 1552.38.

**1.3.7 Synthesis of ZPI-3-N₃ (Scheme 7)**

**Scheme S7.** The synthesis route for ZPI-3-N_3_.

**(1) Synthesis of Compound D2**

Compound D2 was synthesized according to a reported procedure. A 100 mL round-bottom flask was charged with 2-(3-bromophenyl)pyridine (128 mg, 0.55 mmol) and iridium(III) chloride hydrate (69.52 mg, 0.22 mmol) dissolved in 2-methoxyethanol (15 mL) and H₂O (5 mL). The mixture was refluxed at 110 °C under a nitrogen atmosphere for 24 h. After cooling to room temperature, distilled water (15 mL) was added to the reaction mixture, resulting in the formation of a bright yellow precipitate. The solid was collected by filtration, washed thoroughly with water and methanol until the filtrate was colorless, and dried under vacuum to give compound D1 as a yellow solid (105 mg, 61.0% yield). Compound D1 (200 mg, 0.19 mmol) and compound B1 (156 mg, 0.38 mmol) were dissolved in a mixture of dichloromethane/methanol (10 mL/20 mL) and stirred at 55 °C for 8 h. The solvent was removed under reduced pressure, and the residue was purified by silica gel column chromatography using dichloromethane/methanol (v/v = 10:1) as the eluent to afford compound D2 as a yellow powder (253 mg, 62.47% yield). ^1^H NMR (400 MHz，CDCl_3_): δ (ppm) 8.51 (d, *J* = 8.0 Hz, 2H), 8.17 (d, *J* = 4.0 Hz, 2H), 7.94 (d, *J* = 8.0 Hz, 2H), 7.90 - 7.84 (m, 2H), 7.82 - 7.74 (m, 3H), 7.52 (s, 3H), 7.25 (s, 3H), 7.10 (dd, *J* = 8.0, 4.0 Hz, 2H), 7.06 - 6.96 (m, 2H), 6.63 (s, 1H), 6.27 (d, *J* = 8.0 Hz, 2H), 5.32 (s, 1H), 1.5 (s, 9H). MS (MALDI-TOF, [M - Cl]^+^) m/z: calcd for C_46_H_35_Br_2_IrN_7_O_2_: 1068.08. Found: [M - Cl]^+^ 1068.14.

**(2) Synthesis of ZPI-3-N₃**

The synthesis of ZPI-3-N₃ followed a procedure similar to that of ZPI-3. A 50 mL round-bottom flask was charged with ZnPc-COOH (30 mg, 0.042 mmol), D2 (44.86 mg, 0.042 mmol), HATU (80 mg, 0.21 mmol), triethylamine (21 mg, 0.21 mmol), and DMAP (5 mg, 0.042 mmol) in DMF (5 mL). The reaction mixture was stirred at room temperature for 72 h. Additional HATU (40 mg, 0.105 mmol) and DMAP (2.5 mg, 0.021 mmol) were added every 12 h. Upon completion, water was added to the mixture, yielding a blue-green precipitate. The solid was collected by centrifugation, premixed with silica gel, and purified twice by silica gel column chromatography using dichloromethane/methanol (v/v = 30:1) as the eluent to obtain crude D3 as a blue-green solid (12.8 mg).

Compound D3 (20 mg, 0.017 mmol), propargylamine (1.87 mg, 0.034 mmol), Pd₂(dba)₃ (2.75 mg, 0.003 mmol), and KOAc (3.31 mg, 0.034 mmol) were dissolved in DMSO (4 mL) under a nitrogen atmosphere and heated at 90 °C for 12 h. After cooling to room temperature, water was added to precipitate a blue-green solid. The solid was collected by centrifugation, premixed with silica gel, and purified by silica gel column chromatography using dichloromethane/methanol (v/v = 10:1) as the eluent to afford the crude product. The crude product was dissolved in methanol (20 mL), and concentrated hydrochloric acid (3 mL) was added. The mixture was refluxed for 6 h. After completion, saturated aqueous NaHCO₃ solution was added to precipitate a blue solid, which was collected by centrifugation, washed three times with distilled water, and dried under vacuum to yield ZPI-3-N₃ as a blue-green solid (5 mg, 18.2% yield). ^1^H NMR (400 MHz，DMSO-*d_6_*)：δ (ppm) 9.33 (d, *J* = 8.0 Hz, 2H), 9.28 - 9.10 (m, 6H), 9.08 - 9.00 (m, 1H), 8.65 (s, 1H), 8.27 (d, *J* = 8.0 Hz, 2H), 8.23 - 8.16 (m, 6H), 8.16 - 8.07 (m, 4H), 8.03 (dd, *J* = 8.4, 5.2 Hz, 2H), 7.96 (d, *J* = 7.2 Hz, 2H), 7.92 - 7.81 (m ,3H), 7.61 (d, *J* = 8.4 Hz, 2H), 7.51 (d, *J* = 5.2 Hz, 4H), 7.14 (t, *J* = 8.8 Hz, 3H), 7.07 (t, *J* = 7.6 Hz, 2H), 7.02 - 6.90 (m, 5H), 6.73 - 6.62 (m, 4H), 6.30 (d, *J* = 6.8 Hz, 2H), 3.88 (d, *J* = 2.4 Hz, 4H), 3.09 (d, *J* = 2.4 Hz, 2H). MS（MALDI-TOF，[M - Cl]^+^）m/z：calcd for C_86_H_53_IrN_17_O_2_Zn: 1612.35. Found: [M - Cl]^+^ 1614.47.

## 1.4 Cell Viability Assays

Human bladder cancer cell lines T24 and 5637 were cultured in RPMI 1640 medium, the human ureteral epithelial cell line SV-HUC-1 in DMEM/F12K medium, and the murine bladder cancer cell line MB49 in DMEM. MB49 cells were transfected with an mCherry plasmid virus-puro (ATG [711 bp] TAA) to establish the MB49-mCherry cell line. All media were supplemented with 10% newborn bovine serum (NBS), and cells were maintained at 37 °C in a humidified incubator with 5% CO₂.

For cytotoxicity evaluation, T24 and SV-HUC-1 cells were seeded into 96-well plates (2 × 10⁵ cells/well) and cultured for 24 h. Cells were then incubated with serum-free medium containing various concentrations of ZnPc-COOH, Ir-NH₂, ZPI-1, ZPI-2, ZPI-3, ZPI-4, or MMC for 24 h. Cell viability was determined using the CCK-8 assay, and IC₅₀ values were calculated with SPSS software. To validate the activity of ZPI-3, multiple assays (cell counting, EdU-488 incorporation, neutral red cytotoxicity, CCK-8, and MTT) were compared, and the CCK-8 assay was selected for subsequent studies. MB49 cells were treated similarly with ZPI-3, and IC_50_ values were determined.

To evaluate the effect of tetracycline pre‑treatment on ZPI‑3 toxicity, T24 and SV‑HUC‑1 cells were seeded in 96‑well plates (2 × 10^4^ cells/well) and divided into three groups: (1) ZPI‑3 treatment only (ZPI‑3 +; TET −); (2) tetracycline pre‑incubation (0.1 μg/mL, 48 h) followed by combined treatment with tetracycline (0.1 μg/mL) and ZPI‑3 (ZPI‑3 +; TET +); (3) tetracycline pre‑incubation (0.1 μg/mL, 48 h) followed by withdrawal and subsequent ZPI‑3 treatment (ZPI‑3 +; TET +/−). ZPI‑3 concentrations were set at IC_50_ values for each cell line. Cell viability was measured using the CCK‑8 assay.

To investigate differences in the sensitivity of various cell types to ZPI‑3 activity under tetracycline treatment, T24 and SV‑HUC‑1 cells were seeded in 96‑well plates (2 × 10^4^ cells/well) and pre‑incubated with tetracycline at different concentrations (0, 0.01, 0.1 μg/mL) for 48 h. Cells were then treated with ZPI‑3 in the presence of the same tetracycline concentrations for 24 h. Cell viability was measured using the CCK‑8 assay to evaluate the effect of tetracycline pre‑treatment on ZPI‑3 activity.

## 1.5 UV–Vis and Fluorescence Spectroscopy

UV–vis absorption and fluorescence spectra were recorded using quartz cuvettes (0.2 or 1 cm path length) under various experimental conditions.

## 1.6 Subcellular Localization Studies

For intracellular localization, T24 and SV-HUC-1 cells were seeded into glass-bottom dishes (4 × 10⁵ cells/well) and cultured for 24 h. The medium was replaced with RPMI 1640 containing ZPI-3 (2.5 μM), and cells were incubated for another 24 h. Mitochondrial tracker green dye (100 nM) was then added for 2 h. After washing twice with PBS, live-cell imaging was performed using an ECLIPSE Ti-s microscope.

## 1.7 Cellular Uptake Assay

T24 and SV-HUC-1 cells were seeded into 6-well plates (1 × 10⁶ cells/well) and cultured for 24 h. Cells were then incubated with phenol red-free medium containing ZPI-3 (5 μM) for 24 h. Supernatants were collected, and absorbance at 685 nm was measured. A calibration curve was generated using blank supernatants spiked with ZPI-3 (0–5 μM). Residual drug concentrations were determined from the calibration curve, and uptake efficiency was calculated by subtracting the residual concentration from the initial concentration.

## 1.8 Pull-Down Assay

T24 and SV-HUC-1 cells were washed with PBS (4 °C) and lysed in buffer containing 20 mM Tris (pH 7.5), 150 mM NaCl, 1% v/v Triton X-100, and 0.025% v/v protease inhibitor cocktail, followed by incubation on ice for 60 min. Lysates were centrifuged at 12,000 g for 10 min at 4 °C, and protein concentrations were adjusted to 2 mg/mL using a BCA assay. Lysates were incubated with ZPI-3-N₃ (5 μM) at 4 °C for 2 h, followed by a click reaction with 80 μM biotin-N₃, 100 μM TBTA, 1 mM TCEP, and 1 mM CuSO₄ at 20 °C for 1 h. Target proteins were purified using streptavidin magnetic beads (pretreated according to the manufacturer’s instructions). Enriched proteins were eluted with IP lysis buffer containing 0.1% SDS and analyzed by SDS–PAGE with silver staining and Western blotting. Protein bands were excised and subjected to LC–MS/MS analysis.

## 1.9 Competitive Pull-Down Assay

For competitive assays, T24 and SV-HUC-1 lysates (2 mg/mL) were preincubated with ZPI-3 (10 μM) or ZnPc-COOH (10 μM) at 4 °C for 2 h, followed by incubation with ZPI-3-N₃ (5 μM) for another 2 h. A click reaction was performed as described above. Target proteins were purified using streptavidin magnetic beads (Beyotime), eluted with RIPA buffer containing 0.1% SDS, and analyzed by SDS–PAGE with silver staining and Western blotting.

## 1.10 Cellular Thermal Shift Assay (CETSA)

CETSA was performed as described in the literature. Briefly, T24 and SV-HUC-1 cell lysates were prepared as in the pull-down assay and adjusted to 2 mg/mL protein concentration (BCA assay). Lysates were incubated with DMSO or ZPI-3 (10 μM) at 25 °C for 2 h, aliquoted (100 μL each), and heated at 42–72 °C for 5 min. After centrifugation (18,000 g, 20 min, 4 °C), supernatants were analyzed by Western blotting.

For the thermal stability assay of SDHA inhibitors 3-nitropropionic acid (3-NP) and sodium malonate (SM), T24 and SV-HUC-1 cell lysates were prepared according to the pull-down assay protocol, and protein concentrations were adjusted to 2 mg/mL using the BCA method. The lysates (1 mL) were incubated with DMSO (5 μL), SM (0.2 M in H₂O, 5 μL + 5 μL DMSO), and 3-NP acid (0.2 M in DMSO, 5 μL) at 25 °C for 2 hours, aliquoted (100 μL per tube), and then heated at 42 - 72°C for 5 minutes. After centrifugation (18,000 g, 20 minutes, 4 °C), the supernatants were collected for Western blot analysis.

In the experiment examining the effect of different concentrations of ZPI-3 on SDHA protein thermal stability at a fixed temperature, T24 and SV-HUC-1 cell lysates were prepared according to the pull-down assay protocol, and protein concentrations were adjusted to 2 mg/mL using the BCA method. The lysates were incubated with DMSO and varying concentrations of ZPI-3 (10 μM, 20 μM, and 40 μM) at 25 °C for 2 hours (with DMSO content maintained at 0.5% across all groups). The samples were then heated at 67 °C for 5 minutes. After centrifugation (18,000 g, 20 minutes, 4 °C), the supernatants were collected for Western blot analysis.

## 1.11 Co-immunoprecipitation (Co-IP) Assay

Co-IP assays were performed according to the manufacturer’s instructions of the Protein A+G magnetic bead immunoprecipitation kit and published protocols. Briefly, T24 and SV-HUC-1 cells were washed three times with PBS and lysed on ice for 5 min in lysis buffer supplemented with protease inhibitors. Lysates were centrifuged at 12,000 g for 5 min at 4 °C, and the supernatant was collected; 50 μL was reserved as the input sample. Magnetic beads (100 μL) were incubated with antibodies against MIC_60_ or SDHA (20 μg/mL, 500 μL) for 1 h at 25 °C on a rotator to generate antibody-conjugated beads. Cell lysates were incubated with MIC_60_- or SDHA-conjugated beads for 2 h at 25 °C. Beads were separated on a magnetic rack for 30 s, washed three times with lysis buffer containing protease inhibitors, and bound proteins were eluted with SDS-PAGE loading buffer for subsequent Western blotting.

For Co-IP assays detecting acetylated SDHA, deacetylase inhibitors were included in the lysis buffer. T24 and SV-HUC-1 cells were washed three times with PBS and lysed on ice for 5 min in lysis buffer containing both protease and deacetylase inhibitors. Lysates were centrifuged at 12,000 g for 5 min at 4 °C, and protein concentrations were adjusted to 0.6 mg/mL using a BCA protein assay kit. A 100 μL aliquot was reserved as the input sample. Magnetic beads (100 μL) were incubated with anti-SDHA antibody (20 μg/mL, 500 μL) for 1 h at 25 °C to prepare antibody-conjugated beads. Cell lysates were incubated with SDHA-conjugated beads for 2 h at 25 °C, followed by magnetic separation, three washes with lysis buffer containing protease inhibitors, and elution with SDS-PAGE loading buffer for Western blotting.

## 1.12 Western Blot Analysis

Proteins were separated by SDS-PAGE and transferred onto polyvinylidene difluoride (PVDF) membranes. Membranes were blocked with 5% nonfat milk for 1 h, followed by overnight incubation at 4 °C with primary antibodies. After washing three times with TBST (Tris-buffered saline containing 0.1% Tween-20) for 5 min each, membranes were incubated with HRP-conjugated secondary antibodies for 1 h at room temperature. Signals were developed using SuperSignal West Pico chemiluminescent substrate and visualized with a Tanon-5200 Multi imaging system. Quantitative analysis of immunoblot bands was performed using the Gels analysis module in ImageJ software.

## 1.13 MIC_60_ and SDHA Knockdown

Small interfering RNAs (siRNAs) were used to knock down MIC_60_ and SDHA expression in T24 and SV-HUC-1 cells. Cells were transfected with siMIC_60_ (ACTCAGAGGAATCGAACAGGCTG) or siSDHA (GATTTGCTGATGGAAGCATAA) using liposome-mediated transfection.

For stable knockdown, shRNA‑expressing lentiviruses targeting MIC_60_ or SDHA were introduced into T24 and SV‑HUC‑1 cells to generate shMIC_60_-1 and shSDHA-1 lines. A second transduction produced shMIC_60_‑2 and shSDHA‑2 lines. Control lines (shNC T24 and shNC SV‑HUC‑1) were generated using non‑targeting lentiviruses. All cells were maintained at 37 °C in a humidified incubator with 5% CO_2_ in medium containing 10% newborn bovine serum (NBS).

## 1.14 Succinate Dehydrogenase (SDH) Activity Assay

SDH activity in T24 and SV-HUC-1 cells was measured using a commercial kit according to the manufacturer’s instructions.

To investigate the effect of tetracycline on ZPI‑3–mediated regulation of cellular SDH activity, T24 and SV‑HUC‑1 cells were seeded in 6‑well plates (2 × 10^5^ cells/well) and divided into four groups: (1) control (ZPI‑3 −; TET −); (2) ZPI‑3 treatment only (ZPI‑3 +; TET −); (3) tetracycline pre‑incubation (0.1 μg/mL, 72 h; ZPI‑3 −; TET +); (4) tetracycline pre‑incubation (0.1 μg/mL, 48 h) followed by withdrawal and 24 h incubation in drug‑free medium (ZPI‑3 −; TET +/−). ZPI‑3 concentrations were set at IC_50_ values for each cell line (38.5 μM for T24; 275 μM for SV‑HUC‑1). SDH activity was measured using commercial kits, and SDHA and Ac‑SDHA protein levels were assessed by Western blot.

To investigate the effect of deacetylase inhibitors on ZPI-3-regulated SDH activity, the activity of SDH in various cell lines was measured using a commercial assay kit following the manufacturer’s instructions. The cell lines included T24, T24 shSDHA-2, SV-HUC-1, and SV-HUC-1 shSDHA-1. Cells were seeded in 6-well plates and cultured for 24 hours until reaching a density of 1 × 10⁶ cells per well. Crude protein extracts were prepared from each well using cell lysis buffer either with or without deacetylase inhibitors. Subsequently, each group was treated with or without ZPI-3 (5 μM) and incubated for 5 minutes. Following the kit protocol, detection reagents were then added, and SDH activity was measured using a microplate reader.

## 1.15 Intracellular Succinate Measurement

Intracellular succinate levels were measured using a commercial kit. T24 and SV-HUC-1 cells were seeded in 6-well plates and cultured for 24 h until reaching 1 × 10⁶ cells per well. Cells were treated with ZPI-3 (38.5 μM) for 24 h, washed three times with PBS, digested with trypsin, and extracted with n-butanol:methanol:water (5:25:70, v/v/v). Supernatants were collected after centrifugation and analyzed according to the succinate assay kit instructions.

## 1.16 Metabolomics Analysis

T24 cells, SV-HUC-1 cells, and cells treated with ZPI-3 (38.5 μM) were harvested (1 × 10⁷ cells per sample) for metabolomic profiling. After three washes with cold PBS, cells were digested with trypsin, placed on dry ice, and weighed (30 mg/sample). Samples were extracted with 400 μL precooled methanol:acetonitrile (3:1, v/v, −40 °C) containing zirconium oxide beads, homogenized, and incubated at 4 °C for 2 h. After centrifugation (12,000 rpm, 15 min, 4 °C), supernatants were vacuum-dried and reconstituted in 100 μL of 50% methanol. Following vortexing (3 min, 2000 rpm, 4 °C) and centrifugation (12,000 rpm, 15 min, 4 °C), supernatants were subjected to UHPLC analysis.

Samples were maintained at 8 °C in an autosampler and separated on an HSS T3 column using a UHPLC system. Injection volume was 2 μL, column temperature 40 °C, and flow rate 0.3 mL/min. Mass spectrometry was performed on a Thermo QE HF-X instrument with electrospray ionization (ESI) in both positive and negative ion modes. Parameters were: sheath gas flow 30, auxiliary gas 10, spray voltage ±2.5 kV, S-Lens RF 50, capillary temperature 325 °C, auxiliary gas temperature 300 °C, normalized collision energy (NCE) 30, isolation window 1.5 m/z, Top N = 8. The scan range was 70–1050 m/z. Data were acquired using Xcalibur 4.1 software.

## 1.17 Molecular Docking

The crystallographic structure of SDH from Gallus gallus (PDB ID: 2FBW) was obtained from the RCSB Protein Data Bank. Docking analysis was performed using AutoDock Vina. The grid box was centered at x = 42.732, y = −61.059, z = 19.889, with dimensions of 74 × 68 × 82 Å in the x, y, and z directions, respectively. The binding pose with the lowest binding energy was selected, and visualization was conducted using PyMOL (version 2.4.0a0, Schrödinger, LLC).

## 1.18 Mitochondrial Morphology Analysis

Mitochondrial morphology after drug treatment was examined by transmission electron microscopy (TEM). T24, T24 (ZPI-3 treated, 38.5μM for 24 h) and T24 shMIC_60_-2 cells (5 × 10^5^) were harvested with a cell scraper, centrifuged at 3000 rpm for 10 min, and the pellet (~0.05 cm³) was fixed with 2.5% glutaraldehyde at 4 °C overnight. Samples were rinsed three times (15 min each) with 0.1 M phosphate buffer (pH 7.0), post-fixed with 1% osmium tetroxide for 1–2 h, rinsed again, and dehydrated through a graded ethanol series (30%, 50%, 70%, 80%, 90%, and 95%, 15 min each), followed by 100% ethanol (20 min) and pure acetone (20 min). Samples were infiltrated sequentially with embedding resin/acetone mixtures (1:1, 1 h; 3:1, 3 h), then with pure resin overnight, and polymerized at 70 °C for 12 h. Ultrathin sections (~70 nm) were cut, stained with uranyl acetate and lead citrate for 10 min each, air-dried, and examined by TEM.

Live-cell imaging was also performed to assess mitochondrial morphology. T24 and SV-HUC-1 cells (4 × 10⁵ per well) were seeded in confocal dishes, treated with ZPI-3 (2 μM) for 24 h, washed, and incubated with a mitochondrial probe (100 nM) for 2 h. Mitochondrial morphology was observed using an ECLIPSE Ti-s microscope.

## 1.19 Intracellular ATP Measurement

T24 and SV-HUC-1 cells were seeded in 6-well plates (1 × 10⁶ cells/well) and treated with ZPI-3 (38.5 μM or 275 μM) for 24 h. Intracellular ATP levels were determined using an ATP assay kit (Beyotime, China) according to the manufacturer’s instructions.

## 1.20 Intracellular GSH and GSSG Measurement

Intracellular GSH and GSSG levels were measured using a GSH/GSSG assay kit. Standard curves were generated using serial dilutions of 10 mM GSSG standard (0.5–15 μM). T24 and SV-HUC-1 cells (1 × 10⁶/well) were treated with ZPI-3 (38.5 μM or 275 μM) for 24 h, washed with cold PBS, and lysed with protein-removal reagent M. Lysates were centrifuged at 12,000 g for 10 min at 4 °C, and supernatants were collected. For GSSG detection, aliquots were treated with GSH scavenger reagents and incubated at 25 °C for 60 min to remove GSH, followed by total glutathione measurement. For total GSH (GSH + GSSG), 10 μL of supernatant was incubated with 150 μL of working solution for 5 min at room temperature, followed by addition of 50 μL NADPH (0.5 mg/mL). Absorbance at 412 nm was measured after 10 min. GSH content was calculated as: GSH = total glutathione − 2 × GSSG.

## 1.21 Theoretical Calculations

All calculations were performed using Gaussian 16. The molecular structures of ZPI-1 and ZPI-3 were fully optimized at the PBE0-D3BJ/def2-SVP level. Based on the optimized ground-state geometries, time-dependent density functional theory (TD-DFT) was employed to calculate the energies of the lowest singlet (S₁–S₃) and triplet (T₁–T₃) excited states.

## 1.22 Electrochemical Measurements

**(1) Photoelectrochemical experiments.**

Photocurrent responses and charge transfer resistance were measured using a three-electrode system, with glassy carbon electrodes coated with ZPI-1 or ZPI-3 as working electrodes, a platinum electrode as the counter electrode, and an Ag/AgCl electrode as the reference. Measurements were performed in 0.01 M phosphate buffer under 680 nm LED illumination (24 mW/cm²), with light on/off cycles every 30 s.

**(2) Electrochemical impedance spectroscopy (EIS).**

EIS was conducted in an electrolyte containing 0.1 M K₃[Fe(CN)₆], 0.1 M K₄[Fe(CN)₆], and 0.1 M KCl. A three-electrode system was used with a glassy carbon working electrode, platinum counter electrode, and saturated calomel reference electrode. Frequencies ranged from 100 kHz to 100 mHz. ZPI-1 or ZPI-3 was immobilized on the working electrode prior to measurement.

## 1.23 Reactive Oxygen Species (ROS) Detection

DCFH was used to evaluate total ROS generation. DCFH was prepared by hydrolyzing DCFH-DA (4 mM in DMSO, 50 μL) with NaOH (10 mM, 100 μL) for 30 min in the dark, followed by neutralization with PBS (3 mL, pH 7.2). DCFH (0.6 mM, 30 μL) was mixed with PBS (3 mL) and irradiated with 680 nm LED light. Fluorescence intensity of DCF (E_x_: 480 nm, E_m_: 520 nm) was recorded using a fluorescence spectrometer.

For intracellular ROS detection after light exposure, T24 cells (1 × 10⁶/well) were treated with ZPI-1 (7.5 nM) or ZPI-3 (7.5 nM) for 4 h, and then irradiated (680 nm, 12 J/cm²). After irradiation, the cells were further incubated for 20 hours. Subsequently, the cells were washed three times with PBS, and incubated with 10 μM DCFH-DA for 20 min at 37 °C in the dark. After washing, fluorescence images were acquired using an inverted fluorescence microscope (E_x_: 488 nm, E_m_: 500–550 nm). ROS levels were also quantified by flow cytometry.

For the detection of superoxide anions in cellular mitochondria (under dark conditions), T24 and SV-HUC-1 cells were seeded in 6-well plates at a density of 1 × 10⁶ cells per well. After treatment with ZPI-3 (38.5 μM) for 24 hours, cells were washed three times with PBS and incubated with MitoSOX™ Red (5 μM) at 37°C in the dark for 30 minutes. Superoxide anions levels were then quantitatively analyzed using flow cytometry.

For the detection of total intracellular reactive oxygen species (under dark conditions), T24 and SV-HUC-1 cells were seeded in 6-well plates at a density of 1 × 10⁶ cells per well. After treatment with ZPI-3 (38.5 μM) for 24 hours, cells were washed three times with PBS and incubated with DCFH-DA (10 μM) at 37°C in the dark for 4 hours. ROS levels were subsequently quantified using flow cytometry.

## 1.24 Transcriptomic Analysis

T24, SV-HUC-1, and ZPI-3–treated T24 cells (1 × 10⁷ cells/sample) were subjected to RNA sequencing. Total RNA was extracted using Trizol, and purity and integrity were assessed with NanoDrop One and Agilent 2100 or FA 471. mRNA was enriched using VAHTS mRNA Capture Beads, fragmented to 250–450 bp, and reverse-transcribed to cDNA. Double-stranded cDNA was end-repaired, A-tailed, ligated to adapters, purified, and size-selected (250–350 bp). Libraries were amplified by PCR with dual-index primers, purified, and quality-checked using Qubit 4.0, LabChip/FA, and ABI QuantStudio 12K. Sequencing was performed on an Illumina NovaSeq 6000 platform with paired-end reads. Three biological replicates were included per group (n = 3).

## 1.25 Clinical Data and SDH Activity in Clinical Samples

Clinical data of bladder cancer patients were obtained from TCGA (https://portal.gdc.cancer.gov/) and GTEx (https://www.gtexportal.org/home/).

For SDH activity assays, nine clinical samples of non-muscle-invasive bladder cancer (tumor and matched adjacent normal tissues) were analyzed. Approximately 0.05 cm³ of tissue was homogenized in precooled buffer, centrifuged at 11,000 g for 10 min at 4 °C, and protein concentrations were determined using a BCA kit. Supernatants were divided into two groups: untreated control and ZPI-3–treated (1 μM, 30 min). SDH activity was measured according to the kit protocol using a microplate reader.

## 1.26 In Vivo Antitumor Activity

For the drug safety evaluation experiment, healthy C57BL/6 mice were randomly divided into four groups, with 9 mice in each group: PBS control group, NMIBC clinical perfusion drug MMC group, ZPI-3 group, and ZPI-3+Light group. Bladder perfusion was administered on days 1, 5, 9, and 13, with MMC (0.625 mg/kg) and ZPI-3 (0.625 mg/kg). Body weight changes were monitored throughout the experimental period. On day 14, serum samples were collected from three randomly selected mice in each group for biochemical analysis. Additionally, a blinded randomization method was used to select mice for pathological evaluation of major organs and normal bladder tissue.

An orthotopic NMIBC mouse model was established as previously described. Female C57BL/6 mice (~20 g) were used. MB49-mCherry bladder cancer cells, stably expressing luciferase (generated by transfection with an mCherry plasmid virus), were resuspended in 50% mouse serum at a concentration of 2 × 10⁷ cells/mL. Mice were anesthetized with isoflurane, and residual urine was expelled by gentle abdominal pressure. A catheter (0.7 × 19 mm, BD 8234041) was inserted transurethrally, and 5 μL of AgNO₃ (0.3 M) was instilled into the bladder for 15 s to disrupt the urothelium, followed by immediate PBS flushing. Subsequently, 50 μL of cell suspension containing 1 × 10⁶ MB49-mCherry cells was instilled and retained for 1 h. Tumor formation was confirmed by IVIS imaging; mCherry fluorescence was detectable in the bladder region 10 days post-instillation, indicating successful NMIBC model establishment (mice were fasted for 12 h prior to imaging).

To evaluate the administration routes of ZPI-3, both intravesical instillation and intravenous injection (0.3215mg/kg) were tested. Following administration, mice were sacrificed, and the biodistribution of ZPI-3 in bladder tumors and major organs (heart, liver, spleen, lung, kidney, and brain) was assessed by IVIS imaging. To determine whether 680 nm light could penetrate subcutaneous tissue and reach the bladder for PDT, an in vitro ROS generation assay was performed. Two groups were tested: (i) direct irradiation of aqueous solution containing ZPI-3 and DCFH (ROS probe), and (ii) irradiation through excised bladder and subcutaneous tissue. ROS generation was monitored to confirm light penetration.

NMIBC-bearing mice were randomized into four groups (n = 9 per group): PBS (control), MMC (mitomycin C), ZPI-3 (drug only), and ZPI-3 + light (drug combined with PDT). Intravesical instillations were performed on days 0, 7, and 14 after tumor establishment. For the ZPI-3 + light group, laser irradiation (680 nm, 12 J/cm²) was applied to the bladder region 12 h after drug instillation on days 1, 8, and 15. Tumor fluorescence was monitored by IVIS on days 0, 7, 14, and 21, and body weight was recorded. During IVIS imaging, if newly grown hair affects tumor fluorescence imaging, perform additional hair removal treatment on the abdominal hair of the mice. On day 21, four mice from each group were euthanized, and bladders were excised, fixed, embedded, sectioned, and stained for histological analysis.

## 1.27 Microscale Thermophoresis Experiment

The Monolith NT.115 instrument (Nanotemper Technologies) was used to determine the affinity between SDHA protein and ZPI-3. According to the manufacturer’s instructions, ZPI-3 was adjusted to a concentration of 0.5 nM in PBS buffer containing 0.05% Tween, which served as the MST experiment buffer. In each experiment, 10 µL of ZPI-3 (0.5 nM) was mixed with an equal volume of SDHA protein at different concentrations (16 gradient concentrations in total, ranging from 0.01 nM to 450 nM) at room temperature. Subsequently, the samples were loaded into premium capillaries (NanoTemper Technologies) and detected at 25°C using 80% LED power and medium MST power. The data were analyzed using MO. Affinity Analysis v.2.3 software, and the obtained K_d_ values all fell within the valid range of the measurement method.

## 1.28 Statistical Analysis

All experimental data are presented as mean ± standard deviation (mean ± s.d.). Quantitative data between two groups were compared using the t-test; comparisons among multiple groups were performed using one-way analysis of variance (one-way ANOVA). If the between-group differences were statistically significant, further post hoc multiple comparisons were conducted using Tukey’s method. Survival curves were plotted using the Kaplan–Meier method, and the statistical significance of differences between groups was assessed with the log-rank test. All statistical analyses were carried out with GraphPad Prism software, and a P-value < 0.05 was set as the threshold for statistical significance. Significance levels are denoted as: *p < 0.05, **p < 0.01, ***p < 0.001; n.s. indicates no statistically significant difference.

# 2 Supplementary Data and Figures

Mitochondria-targeted drugs may interfere with mitochondrial function, thereby affecting the accuracy of cell viability assays. To address this concern, we evaluated the cytotoxicity of ZPI-3 toward T24 cells using multiple viability assays, including MTT, CCK-8, neutral red uptake, EdU incorporation, and direct cell counting methods.^1-3^ Consistent results were obtained across all methods except for the MTT assay (Figure S1). Therefore, the CCK-8 assay, which is relatively simple and reliable, was chosen for subsequent activity studies.

**Figure S1.** Comparison of T24 cell cytotoxicity of ZPI-3 (5 μM) measured by multiple cell viability assays, including MTT, CCK-8, neutral red, EdU incorporation, and cell counting. Data are presented as the mean ± s.d. (n = 3, each group). Group differences were analysed using one-way ANOVA followed by multiple comparisons. n.s., not significant, ***p < 0.001.

In aqueous solution, the UV–vis absorption spectra of ZnPc-COOH, Ir-NH₂, and ZPI-1～ZPI-4 gradually increased with increasing drug concentration.

**Figure S2.** UV–vis absorption spectra of ZnPc-COOH (<150 μM), Ir-NH_2_ (<200 μM), and ZPI-1～ZPI-4 (<300 μM) in aqueous solution as a function of concentration (cuvette path length = 2 mm).

**Figure S3.** Linear ranges of absorbance versus concentration for ZnPc-COOH (685 nm), Ir-NH_2_ (270 nm), and ZPI-1～ZPI-4 (685 nm) in aqueous solution (cuvette path length = 2 mm).

In the range of 500～800 nm, ZPI-3 exhibited characteristic phthalocyanine absorption peaks. Upon excitation at 647 nm (corresponding to the laser wavelength equipped on the confocal microscope), ZPI-3 displayed strong fluorescence emission in the near-infrared region (Figure S4). Therefore, confocal laser scanning microscopy was employed to visualize its intracellular distribution. In both T24 and SV-HUC-1 cells, ZPI-3 was localized in the mitochondria, with Pearson’s correlation coefficients of 0.88 and 0.81, respectively (Figure 2A and Figure S5). The selective cytotoxicity of ZPI‑3 in T24 cells does not arise from variations in intracellular distribution between tumor and normal cells.

**Figure S4.** UV-vis absorption spectrum (A) and fluorescence emission spectrum (B) of ZPI-3 (cuvette path length = 1 cm).

**Figure S5.** Localization of ZPI-3 (2.5 μM) in T24 and SV-HUC-1 cells after 24 h of treatment.

**Figure S6.** Molecular structure of ZPI-3-N_3_ (A) and comparison of dark cytotoxicity between ZPI-3-N_3_ and ZPI-3 in T24 cells (B).

**Figure S7.** Schematic diagram of the competitive pulldown assay.

**Figure S8.** Effect of ZPI-3 (10 μM) on the thermal stability of HSPA9, AIFM1, NDUFS1, and ACADVL in T24 cells.

**Figure S9.** Effect of ZPI-3 (10 μM) on the thermal stability of HSPA9, AIFM1, NDUFS1, and ACADVL in SV-HUC-1 cells.

**Figure S10.** Effects of SM and 3‑NP on the thermal stability of SDHA in T24 cells (A) and SV‑HUC‑1 cells (B), measured by CETSA. All data are presented as the mean ± s.d. (*n* = 3, each group). Group differences were analysed by the two-tailed Student’s *t* test. *p < 0.05, **p < 0.01, ***p < 0.001.

The potency of drug-protein binding can be assessed using an isothermal dose-response CETSA (ITDR-CETSA) experiment. In this method, a fixed temperature is applied across a range of drug concentrations to monitor dose-dependent thermal stabilization ^4, 5^. The temperature is typically set at a point where most of the unliganded target protein is denatured and precipitated ^6^. Therefore, we selected 67°C for the ITDR-CETSA experiment to evaluate the binding of ZPI-3 to SDHA in T24 cells. As shown in Figure S11, the ITDR-CETSA results indicate that as the concentration of ZPI-3 increases, the thermal stability of SDHA gradually decreases, further confirming that ZPI-3 can bind to SDHA.

**Figure S11.** Effects of different concentrations of ZPI‑3 on the thermal stability of SDHA in T24 cells, measured by ITDR‑CETSA. All data are presented as the mean ± s.d. (n = 3, each group).

**Figure S12.** (A) Differences in proliferation between T24 WT and T24 shNC-G cells (A1) and ZPI-3 activity in the two cell lines (A2); (B) Differences in proliferation between SV-HUC-1 WT and SV-HUC-1 shNC-G cells (B1) and ZPI-3 activity in the two cell lines (B2). Data are presented as the mean ± s.d. (n = 3, each group). Group differences were analysed using two-tailed Student’s t test. n.s., not significant.

**Figure S13.** (A) Differences in proliferation between T24 WT and T24 shNC-R cells (A1) and ZPI-3 activity in the two cell lines (A2); (B) Differences in proliferation between SV-HUC-1 WT and SV-HUC-1 shNC-R cells (B1) and ZPI-3 activity in the two cell lines (B2). Data are presented as the mean ± s.d. (n = 3, each group). Group differences were analysed using two-tailed Student’s t test. n.s., not significant.

**Figure S14.** (A) Effect of varying degrees of MIC_60_ knockdown on MIC_60_ expression in T24 cells: blotting (A1) and quantitative analysis (A2); impact on T24 cell proliferation (A3) and ZPI-3 activity (A4). (B) Effect of varying degrees of MIC_60_ knockdown on MIC_60_ expression in SV-HUC-1 cells: blotting (B1) and quantitative analysis (B2); impact on SV-HUC-1 proliferation (B3) and ZPI-3 activity (B4). Data are presented as the mean ± s.d. (n = 3, each group). Group differences were analysed using two-tailed Student’s t test, n.s., not significant *p < 0.05, **p < 0.01, ***p < 0.001.

**Figure S15.** Protein levels of SDHA and MIC_60_ in T24 shSDHA/shMIC_60_ cells. Data are presented as the mean ± s.d. (n = 3, each group). Group differences were analysed using two-tailed Student’s t test. ***p < 0.001.

**Figure S16.** Changes in ZPI-3 activity and effects on T24 cell proliferation in cells with combined knockdown of SDHA and MIC_60_ (T24 shSDHA/shMIC_60_). Data are presented as the mean ± s.d. (n = 3, each group). Group differences were analysed using two-tailed Student’s t test. n.s., not significant.

**Figure S17.** Transmission electron microscopy images of T24 and T24 shMIC_60_‑2 cell sections. ^a^ Image brightness was increased by 20% and contrast by 40% to more clearly visualize mitochondrial onion‑like cristae structures.

**Figure S18.** Changes in ATP synthesis levels in T24 shMIC_60_-1 and T24 shMIC_60_-2 cells. All data are presented as mean ± standard deviation (n = 3 per group). Differences between groups were analyzed using a two-tailed Student’s t-test, with n.s. indicating no significant difference, and ***p < 0.001.

**Figure S19.** Effect of SDHA knockdown on the proliferation of SV-HUC-1 (A) and T24 (B) cells. Group differences were analysed using two-tailed Student’s t test. *p < 0.05，**p < 0.01.

**Figure S20.** Blotting of SDHA in T24 and SV-HUC-1 cells (A) and comparison of protein levels (B). Data are presented as the mean ± s.d. (n = 3, each group). Group differences were analysed using two-tailed Student’s t test. n.s., not significant.

**Figure S21.** Comparison of SDH activity in T24 WT, T24 shSDHA-2, SV-HUC-1, and SV-HUC-1 shSDHA-1 cells. Data are presented as the mean ± s.d. (n = 3, each group). Group differences were analysed using two-tailed Student’s t test. n.s., not significant.

In the classical MST approach, the protein was fluorescently labeled using the MO-L018 His-Tag Labeling Kit RED-tris-NTA 2^nd^ Generation kit. For each assay, the labeled protein was mixed with an equal volume of unlabeled compounds at various serial concentrations. According to the manual of the MO-L018 His-Tag Labeling Kit RED-tris-NTA 2^nd^ Generation, the dye exhibits fluorescence excitation and emission maxima at approximately 650 nm and 670 nm, respectively, which is very close to the fluorescence signal of ZPI-3. Therefore, we directly utilized the intrinsic fluorescence signal of ZPI-3 to detect its molecular interaction with unlabeled protein (Figure S22).

**Figure S22.** MST analysis of the binding affinity between ZPI‑3 and SDHA protein. A fixed concentration of ZPI‑3 (0.5 nM) was titrated with increasing concentrations of purified recombinant SDHA (0.01 - 450 nM).

**Figure S23.** Effects of deacetylase inhibitors on the ability of ZPI‑3 (5 μM, 30 min incubation) to downregulate SDH catalytic activity in lysates from T24, T24 shSDHA‑2, SV‑HUC‑1, and SV‑HUC‑1 shSDHA‑1 cells. Data are presented as mean ± s.d. (n = 3 per group). Group differences were analyzed using one‑way ANOVA followed by multiple comparisons. n.s., not significant; *p < 0.05; **p < 0.01; ***p < 0.001.

SDH activity is regulated by acetylation, with SIRT3 acting as a key deacetylase that suppresses SDHA acetylation. Myc promotes SDHA acetylation and inactivation by inducing SKP2‑mediated degradation of SIRT3 (Figure S24A). Genomic analyses revealed marked differences in the expression of these regulators between T24 and SV‑HUC‑1 cells (Figure S24B).

SDH comprises four core subunits (SDHA, SDHB, SDHC, and SDHD) and several accessory factors. During succinate oxidation by SDHA, electrons reduce FAD and are transferred to the Fe–S clusters in SDHB. Together with SDHC and SDHD, SDHB then delivers the electrons to ubiquinone, completing a critical step in the mitochondrial electron transport chain (ETC). Genes encoding SDH subunits and assembly factors also showed significant expression differences between T24 and SV‑HUC‑1 cells (Figure S24B).

It should be noted, however, that protein levels and activities are not determined solely by gene expression. Transcriptional, translational, and post‑translational regulation also play crucial roles. Thus, the distinct mechanisms governing SDHA homeostasis and its resistance to perturbation in T24 versus SV‑HUC‑1 cells warrant further investigation.

**Figure S24.** (A) Mechanism of SDHA acetylation regulation and differences in expression of related genes between T24 and SV-HUC-1 cells; (B) heatmap showing gene expression differences related to acetylation regulation, subunits, and complex assembly factors affecting SDH activity in the two cell types.

**Figure S25.** Pull‑down results of ZPI‑3‑N_3_ and competitive pull‑down immunoblot analysis following pre‑treatment with ZPI‑3 or ZnPc‑COOH. All data are presented as the mean ± s.d. (n = 3, each group). Group differences were analysed by the two-tailed Student’s t test. n.s., not significant, ***p < 0.001.

**Figure S26.** Preferred binding sites of ZPI-1, ZPI-2, and ZPI-3 on SDH.

**Figure S27.** Molecular structures of succinic acid, trinitropropionic acid, itaconic acid, citraconic acid, and sodium malonate.

Mitochondria are the central hub of cellular metabolism. SDH is a classic “moonlighting” enzyme, functioning both as the catalyst for succinate oxidation to fumarate in the tricarboxylic acid (TCA) cycle and as complex II in the mitochondrial respiratory chain.^7^ The mitochondrial contact site and cristae organizing system (MICOS) is located at crista junctions and maintains crista architecture, with MIC_60_ as a key structural component. Loss of MIC_60_ disrupts crista organization and produces onion‑like mitochondrial structures.^8^ Respiratory chain proteins, including SDH, and ATP synthase, are distributed along the cristae structure, supporting oxidative phosphorylation (OXPHOS) and the TCA cycle to maintain metabolic homeostasis (Figure S28A1).^9^

Cellular metabolic homeostasis is essential for maintaining redox balance. Since both SDH and MIC_60_ play crucial roles in metabolic stability, we asked whether their disruption could impair redox homeostasis and thereby enhance oxidative damage induced by photodynamic therapy (PDT).^10^

Transmission electron microscopy showed that ZPI‑3 treatment partially transformed T24 cell mitochondria from tubular to onion‑like morphology. Confocal microscopy further revealed that untreated T24 cells displayed normal filamentous mitochondrial networks, whereas ZPI‑3 treatment produced punctate structures (Figure S28A2). A similar, though less pronounced, effect was observed in SV‑HUC‑1 cells (Figure S28A2). These findings indicate that ZPI‑3 disrupts mitochondrial cristae organization, thereby impairing mitochondrial fusion and the formation of filamentous networks.

Oxidative phosphorylation involves two tightly coupled processes: (1) generation of the electrochemical proton gradient (Δ~μH⁺) across the crista membrane by the mitochondrial respiratory complexes, which establishes the mitochondrial membrane potential, and (2) synthesis of ATP by ATP synthase in a membrane potential–dependent manner (Figure S28A1).^11^

**Figure S28.** (A) Schematic illustration of MICOS damage on mitochondrial structure and function (A1) and TEM and confocal images of mitochondrial structure in T24 cells after treatment with ZPI-3 (38.5 μM) for 24 h, as well as confocal images in SV-HUC-1 cells (A2). (B) Comparison of TCA cycle activity (B1) mitochondrial membrane potential (B2, using RH-123) and ATP levels (B3) in T24 and SV-HUC-1 cells after treatment with ZPI-3 (38.5 μM) for 24 h. Flow cytometric detection of mitochondrial superoxide anion (C1) and MitoSO^TM^ Red fluorescence intensity (C2) in T24 and SV-HUC-1 cells treated with ZPI-3 (38.5 μM) for 24 h. Intracellular total ROS production (C3) and DCF fluorescence intensity (C4) were measured under the same conditions. Data are presented as the mean ± s.d. (n = 3, each group). Group differences were analysed using two-tailed Student’s t test. n.s., not significant, *p < 0.05, **p < 0.01, ***p < 0.001.

**Figure S29.** KEGG pathway analysis of metabolomic changes in SV-HUC-1 cells after ZPI-3 treatment under positive and negative ion modes.

The photochemical mechanism of PDT involves excitation of the photosensitizer from its ground singlet state (S_0_) to an excited singlet state (S_n_) upon light irradiation. The excited singlet state can undergo intersystem crossing (ISC) to generate a triplet excited state (T_n_), which transfers electrons or energy to surrounding biomolecules or ground-state oxygen molecules to produce reactive oxygen species (ROS) (Figure S30A).^12^

Because the S_1_ state of a photosensitizer is inherently unstable, some molecules return to the ground state (S_0_) through fluorescence emission, thereby reducing the population available for ISC. At the same concentration, ZPI-3 exhibited lower fluorescence intensity than ZPI-1 (Figure S30B), suggesting that fewer excited singlet-state molecules of ZPI-3 decay radiatively and that ZPI-3 possesses a higher ISC efficiency compared to ZPI-1.

To further investigate their charge transfer behaviors, electrochemical impedance spectroscopy (EIS) and photocurrent response experiments were conducted. ZPI-3 showed markedly lower charge‑transfer resistance than ZPI-1 (Figure S30C), indicating faster charge migration in ZPI-3. Consistently, under identical conditions, ZPI-3 generated a pronounced photocurrent, whereas the photocurrent of ZPI-1 was negligible (Figure S30D), confirming that ZPI-3 possesses superior electron transfer capability. The enhanced ISC efficiency and stronger electron transfer ability of ZPI-3 are expected to contribute to its higher PDT activity.^13^

**Figure S30.** (A) Schematic of ROS generation mechanism by photosensitizer excitation. (B～D) Comparison of fluorescence spectra (B), charge-transfer resistance (C), and photocurrent response (D) between ZPI-3 and ZPI-1.

**Figure S31.** ROS generation by ZPI-3 (A) and ZPI-1 (B) in aqueous solution detected by DCFH oxidation, and comparison of activity (C).

**Figure S32.** mCherry-MB49 cells obtained by transfecting MB49 cells with the mCherry gene; fluorescence images (A) and intensity comparison (B) of mCherry protein in MB49 and mCherry-MB49 cells. Group differences were analysed using two-tailed Student’s t test. ***p < 0.001.

**Figure S33.** Isolation of mouse bladder epithelial cells and verification using epithelial marker UPIb; blotting of UPIb in primary bladder epithelial cells and intact bladder tissue (A) and comparison of expression levels (B). Group differences were analysed using two-tailed Student’s t test. **p < 0.01.

**Figure S34.** Comparison of Ac-SDHA levels in mouse primary bladder epithelial cells, MB49, and mCherry-MB49 cells. Data are presented as the mean ± s.d. (n = 3, each group). Group differences were analysed using two-tailed Student’s t test. n.s., not significant, **p < 0.01.

Healthy C57BL/6 mice were divided into four groups: control (PBS), MMC group (clinical instillation drug for NMIBC), ZPI-3 group, and ZPI-3+Light group. Drugs were administered via intravesical instillation on days 1, 5, 9, and 13. Throughout the experiment, changes in mouse body weight were monitored. On day 14, serum biochemical parameters were measured in all groups, and a blinded random selection of mice was performed for pathological evaluation of major organs and normal bladder tissue. The aim was to demonstrate drug safety and establish a dosing rationale under conditions of high instillation frequency (once every 4 days, for a total of 4 instillations) and high instillation dose (0.6250 mg/kg per instillation).

During the 14-day period, no body weight loss or significant differences in weight changes were observed among any of the groups (Figure S35). After treatment with higher instillation frequency and larger doses, no significant changes were found in the major serum biochemical parameters of mice across all groups (Figure S36), and no obvious morphological alterations were observed in histological sections of major organs or normal bladder tissue (Figure S37 and S38). These results preliminarily indicate that both MMC and ZPI-3 exhibited favorable safety profiles under conditions of higher instillation frequency and larger doses. In subsequent in vivo anti-tumor activity studies, a lower instillation frequency (3 times, once every 7 days) and a lower instillation dose (0.3125 mg/kg) were used, allowing anti-tumor efficacy studies to be conducted while ensuring safety. Thus, these supplementary data also provide a dosing basis for the anti-tumor activity research.

**Figure S35.** Comparison of body weight among groups (A) and changes in body weight during the study (B) (drug dose: 0.6250 mg/kg); All animal experimental data are presented as the mean ± s.d. (n = 9, each group). Group differences were analysed using one-way ANOVA followed by multiple comparisons. n.s., not significant.

**Figure S36.** Serum biochemical parameters in mice during the safety study (Phosphorus: P II; Calcium: Ca; Glucose: Glu-G; Direct Bilirubin: T-bil-D II; Total Bile Acids: TBA; Alanine Aminotransferase: ALT; Aspartate Aminotransferase: AST; C-Reactive Protein: CRP II; Alkaline Phosphatase: ALP; γ-Glutamyl Transferase: γ-GT; Total Protein: TP II; Albumin: ALB II; α-Amylase: α-AMY; Triglyceride: TG; Total Cholesterol: TC; Creatinine: CREA-S; Urea Nitrogen: UREA; Creatine Kinase: CK)。Data are presented as the mean ± s.d. (n = 3, each group). Group differences were analysed using one-way ANOVA followed by multiple comparisons, n.s., not significant.

**Figure S37.** Representative H&E‑stained sections of major organs from mice in the safety study.

**Figure S38.** Representative H&E‑stained sections of bladder from mice in the safety study.

Anatomically, the mouse bladder lies subcutaneously in the lower abdomen. As shown in Figure S28, 680 nm light penetrates the bladder and overlying skin, exciting ZPI-3 within the cuvette to generate ROS, which oxidize DCFH into fluorescent DCF. Thus, during in vivo therapy, external irradiation efficiently traverses biological tissues to reach the bladder, activating the PDT-based synergistic effect of ZPI-3.

**Figure S39.** Schematic of experimental setup simulating light penetration to activate ZPI-3 PDT in the mouse bladder (A) and comparison of ZPI-3 ROS generation before and after light penetration through mouse skin and bladder (B).

At day 21, persistent mCherry fluorescence in the bladder indicated residual tumor presence. The proportions of mice without detectable bladder fluorescence at day 21 were 0%, 11.1%, 22.2%, and 77.8% in the control, MMC, ZPI‑3, and ZPI‑3 plus Light groups, respectively (Figure S40). Given the high recurrence rate of NMIBC, the markedly improved outcome in the ZPI‑3 plus Light group provides a promising basis for reducing recurrence and extending survival. It should be noted, however, that evaluating therapeutic efficacy solely by bladder fluorescence intensity is not fully accurate, as the sensitivity of fluorescence detection instruments may be insufficient to identify signals from small residual lesions.

**Figure S40.** Comparison of cure rates in each group of mice on day 21.

All SDH activity assays in this study were conducted using a commercial kit. The principle is as follows: after 24 h co-incubation with ZPI-3, the drug enters mitochondria and binds SDH. Cells are lysed to obtain protein solutions containing SDH, to which succinate, 5-methylphenazinium methosulfate (PMS), and 2,6-dichloroindophenol sodium salt hydrate (DCIP) are added. SDH catalyzes succinate oxidation to fumarate, PMS transfers electrons, and DCIP is reduced, leading to a decrease in its absorption peak at 600 nm. Thus, SDH activity is inversely proportional to residual DCIP. ^14^

To assess ZPI-3 effects on SDH activity in tumor and adjacent normal bladder tissues, primary culture would be required, but this is complex and may compromise accuracy. Based on the kit’s detection principle, we optimized the protocol: after lysis, ZPI-3 was added directly to protein solutions and incubated for 30 min before standard assay steps. In this modified protocol, ZPI-3 still binds SDHA and modulates SDH activity. This approach extends the kit’s applicability to cell and tissue lysates (Figure 7A). Using this method, we detected differential effects of ZPI-3 on SDH activity in tumor and adjacent normal tissues from eighteen NMIBC patients (Figure S30).

**Figure S41.** Individual-patient data comparison of ZPI‑3 effects on SDH activity in paired bladder tumor and peritumoral tissues from 18 NMIBC patients.

ZPI‑3 displayed potent cytotoxic activity in 5637 cells, similar to T24 cells (IC_50_ = 29.03 μM), and effectively downregulated SDH activity. In contrast, ZPI‑3 exhibited poor cytotoxicity in UM‑UC‑3 cells (IC_50_ = 171.74 μM) and failed to significantly reduce SDH activity (Figure S42), resembling the activity profile observed in SV‑HUC‑1 cells.

**Figure S42.** Effects of ZPI‑3 (5 μM, 30 min incubation) to downregulate SDH catalytic activity in lysates from T24, SV‑HUC‑1, 5637, and UM‑UC‑3 cells. Data are presented as the mean ± s.d. (n = 3, each group). Group differences are analysed by the two-tailed Student’s t test. n.s., not significant, ***p < 0.001.

We hypothesized that UM‑UC‑3 cells may share a strong dependency on SDHA similar to SV‑HUC‑1. To test this, we attempted SDHA knockdown in UM‑UC‑3 cells. Transfection was successful, as indicated by mCherry fluorescence in both UM‑UC‑3 shNC and UM‑UC‑3 shSDHA‑1 cells. However, while UM‑UC‑3 shNC cells remained viable, UM‑UC‑3 shSDHA‑1 cells rapidly shrank and died (Figure S43). These results demonstrate that SDHA is essential for UM‑UC‑3 cell survival, consistent with the phenomenon observed in SV‑HUC‑1 cells.

**Figure S43.** Morphology and mCherry fluorescence images of UM‑UC‑3, UM‑UC‑3 shNC, and UM‑UC‑3 shSDHA‑1 cells. Fluorescence image brightness was increased by 40% to enhance visualization.

The Cancer Genome Atlas (TCGA) provides large-scale genomic data to advance cancer prevention, diagnosis, and treatment. ^15^ Among 406 bladder cancer samples in TCGA, only 19 include paired tumor and adjacent normal tissues. As shown in Figure S44A, analysis of these pairs revealed no significant difference in SDHA levels. Moreover, GEPIA2 analysis indicated that SDHA expression was not significantly associated with disease-free survival (Figure S44B) or overall survival (Figure S44C).

These findings suggest that public datasets have not identified SDHA as a driver of bladder cancer or a therapeutic biomarker. However, our study demonstrates that differences between bladder cancer and normal cells lie not in SDHA expression, but in SDH activity, which depends on the deacetylated active form of SDHA. Research on SDHA regulation remains limited, but with deeper insights into the molecular features and regulatory mechanisms of its active form, SDHA activity may emerge as a critical driver of bladder cancer progression and a promising target for precision therapy.

**Figure S44.** (A) Comparison of SDHA levels in 19 paired bladder cancer samples from TCGA, Data are presented as the mean ± s.d. (n = 19, each group). Group differences were analysed using two-tailed Student’s t test. n.s., not significant; (B, C) analysis using GEPIA2 of the impact of SDHA expression on disease-free survival (B) and overall survival (C) of bladder cancer patients in TCGA. Survival curves were plotted using the Kaplan–Meier method, and the statistical significance of differences between groups was assessed with the log-rank test.

**Figure S45.** Schematic overview of the validation and exclusion workflow for potential ZPI‑3 targets in this study.

**Table S1.** The dark IC_50_ and SI value of various drugs in this research.

| **Drugs** | **Dark IC_50_ to SV-HUC-1**  **IC_50-HUC-SV-1_** | **Dark IC_50_ to T24**  **IC_50-T24_** | **SI**  **SI =** **IC_50-HUC-SV-1_/** **IC_50-T24_** |
| --- | --- | --- | --- |
| ZnPc-COOH | 2.39 μM | 5.75 μM | 2.41 |
| Ir-NH_2_ | 0.71 μM | 1.63 μM | 2.29 |
| ZPI-1 | 131.27 μM | > 275 μM | 2.09 |
| ZPI-2 | 127.83 μM | 247.16 μM | 1.93 |
| ZPI-3 | 38.54 μM | > 275 μM | 7.14 |
| ZPI-4 | 37.02 μM | 181.53 μM | 4.90 |
| MMC | 0.79 μM | 2.84 μM | 3.60 |

**Table S2.** The details of pull-down proteins by ZPI-3-N_3_ (Coverage > 20; Unique Peptides > 10).

| **Accession** | **Gene** | **Coverage [%]** | **Unique Peptides** | **MW [kDa]** | **Abundance: F1: Sample** |
| --- | --- | --- | --- | --- | --- |
| P28331 | NDUFS1 | 20 | 12 | 79.4 | 40875412.25 |
| B9A067 | MIC_60_ | 20 | 13 | 78.9 | 63126655.25 |
| P31040 | SDHA | 22 | 13 | 72.6 | 78117355.88 |
| P49748 | ACADVL | 28 | 17 | 70.3 | 155988658.4 |
| O95831 | AIFM1 | 26 | 11 | 66.9 | 77159743 |
| A0A7I2V2G2 | HSPA9 | 49 | 33 | 66.6 | 770229484.9 |

**Table S3.** Nomenclature of T24 and SV-HUC-1 cells with different levels of MIC_60_ or SDHA knockdown, as well as cells with dual SDHA and MIC_60_ knockdown. Note：“shNC‑R” and “shNC‑G” denote control cell lines transduced with lentiviral vectors lacking SDHA or MIC_60_ knockdown, respectively.

| **Original cell** | **Cell name** | **SDHA knock-down level** | **MIC_60_ knock-down level** |
| --- | --- | --- | --- |
| T24 | T24 shSDHA-1 | 70% | 0% |
|  | T24 shSDHA-2 | 90% | 0% |
|  | T24 shNC-R | 0% | 0% |
|  | T24 shMIC_60_-1 | 0% | 63% |
|  | T24 shMIC_60_-2 | 0% | 90% |
|  | T24 shNC-G | 0% | 0% |
|  | T24 shSDHA/shMIC_60_ | 90% | 81% |
| SV-HUC-1 | SV-HUC-1 shSDHA-1 | 67% | 0% |
|  | SV-HUC-1 shNC-R | 0% | 0% |
|  | SV-HUC-1 shMIC_60_-1 | 0% | 68% |
|  | SV-HUC-1 shMIC_60_-2 | 0% | 90% |
|  | SV-HUC-1 shNC-G | 0% | 0% |

**Table S4.** Figures 3D3 and S28B1 involve metabolomics data.

| **Cell line** | **ZPI-3** | **Substrates** | **Average _Normal Area_** | **SD** | **Foldchange**  **ZPI-3 treated VS. untreated** | **Log_2_Foldchange**  **ZPI-3 treated VS. untreated** | **P value** | **P.adjsut** |
| --- | --- | --- | --- | --- | --- | --- | --- | --- |
| T24 | - | α-Ketoglutarate | 634484494.3503 | 69220301.2823 | / | / | / | / |
|  | - | Citrate | 123788050.9729 | 37597077.6866 | / | / | / | / |
|  | - | Fumarate | 809936221.4775 | 32832987.8515 | / | / | / | / |
|  | + | α-Ketoglutarate | 311185257.2608 | 57280966.8569 | -2.3077 | -1.2065 | 0.0074 | 0.0382 |
|  | + | Citrate | 168567972.9317 | 6788784.4680 | -3.4776 | -1.7981 | 0.0331 | 0.1224 |
|  | + | Fumarate | 313570076.9503 | 35197128.7228 | -1.2464 | -0.3178 | 0.0082 | 0.0406 |
| SV-HUC-1 | - | α-Ketoglutarate | 24341905.1285 | 31635066.1245 | / | / | / | / |
|  | - | Citrate | 16606139.6977 | 6901275.4452 | / | / | / | / |
|  | - | Fumarate | 222281408.2540 | 77633397.7814 | / | / | / | / |
|  | + | α-Ketoglutarate | 184183348.4283 | 49991269.7702 | 16.1688 | 4.0151 | 0.0018 | 0.0076 |
|  | + | Citrate | 60817719.8461 | 10552692.2928 | 4.5877 | 2.1978 | 0.0113 | 0.0328 |
|  | + | Fumarate | 359766730.6805 | 34134889.7187 | 1.9805 | 0.9859 | 0.0117 | 0.0334 |

# 3 Reference

[1] R. Podgórski, M. Wojasiński, T. Ciach, *Sci. Rep.* **2022**, *12*, 9047.

[2] G. Repetto, A. Del Peso, J. L. Zurita, *Nat. protoc.* **2008**, *3*, 1125-1131.

[3] Y. Qi, L. Ding, S. Zhang, S. Yao, J. Ong, Y. Li, H. Wu, P. Du, *Cell* **2022**, 185, 1888-1904.

[4] H. Du, T. Xu, S. Yu, S. Wu, J. Zhang, *Signal Transduct. Tar.* **2025**, *10*, 245.

[5] H. Franken, T. Mathieson, D. Childs, G. M. A Sweetman, T. Werner, I. Tögel, C. Doce, S. Gade, M. Bantscheff, G.Drewes, *Nat. Protoc.* **2015**, *10*, 1567-1593.

[6] D. Martinez Molina, P. Nordlund, *Annu. Rev. Pharmacol.* **2016**, *56*, 141-161.

[7] Y. Tu, L. Tan, H. Tao, Y. Li, H. Liu, *Phytomedicine* **2023**, *116*, 154862.

[8] C. Glytsou, E. Calvo, S. Cogliati, A. Mehrotra, I. Anastasia, G. Rigoni, A. Raimondi, N. Shintani, M. Loureiro, J. Vazquez, *Cell Rep.* **2016**, *17*, 3024-3034.

[9] L. Sainero-Alcolado, J. Liaño Pons, M. V. Ruiz Pérez, *Cell Death Differ.* **2022**, *29*, 1304-1317.

[10] Q. Cheng, W. Yu, J. Ye, M. Liu, W. Liu, C. Zhang, C. Zhang, J. Feng, X. Z. Zhang, *Biomaterials* **2019**, *224*, 119500.

[11] I. Martínez-Reyes, N. S. Chandel, *Nat. Commun.* **2020**, *11*, 102.

[12] M. Jia, Y. Pan, W. Hu, *Small Methods* **2025**, e01279.

[13] K.X. Teng, L. Y. Niu, Q. Z. Yang, *J. Am. Chem. Soc.* **2023**, *145*, 4081-4087.

[14] Jonathan G.Van Vranken, Daniel K. Bricker, N. Dephoure, Steven P. Gygi, James E. Cox, Carl S. Thummel, J. Rutter, *Cell Metab.* **2014**, *20*, 241-252.

[15] H. Fu, X. Mo, A. A. Ivanov, *Nat. Rev. Cancer* **2025**, *25*, 189-208.

**Original blot documentation**

**Figure 2. F**

**Figure 2. G**

**Figure 2. H**

**Figure 2. I1**

**Figure 3. A1**

**Figure 3. E1**

**Figure 3. F1**

**Figure 6. A1**

**Figure S8.**

**Figure S9.**

**Figure S10.**

**Figure S11.**

**Figure S14.**

**Figure S15.**

**Figure S20.**

**Figure S25.**

**Figure S33.**
